# Obesity and survival in advanced non-small cell lung cancer patients treated with chemotherapy, immunotherapy, or chemoimmunotherapy: a multicenter cohort study

**DOI:** 10.1186/s12916-024-03688-2

**Published:** 2024-10-14

**Authors:** Wei Nie, Jun Lu, Jie Qian, Shu-Yuan Wang, Lei Cheng, Liang Zheng, Guang-Yu Tao, Xue-Yan Zhang, Tian-Qing Chu, Bao-Hui Han, Hua Zhong

**Affiliations:** 1 Department of Respiratory and Critical Care Medicine, Shanghai Chest Hospital, School of Medicine, Shanghai Jiao Tong University, Shanghai, China; 2 Department of Emergency Medicine, Shanghai Chest Hospital, School of Medicine, Shanghai Jiao Tong University, Shanghai, China; 3 Department of Radiology, Shanghai Chest Hospital, School of Medicine, Shanghai Jiao Tong University, Shanghai, China

**Keywords:** Body mass index, Obesity, Non-small cell lung cancer, Survival

## Abstract

**Background:**

The association of body mass index (BMI) with survival outcomes in patients with advanced non-small cell lung cancer (NSCLC) treated with first-line chemotherapy, immunotherapy, or chemoimmunotherapy is controversial. We aimed to investigate these associations, including associations in male and female patients specifically, in a multicenter cohort study.

**Methods:**

We retrospectively analyzed data from seven cohorts comprising 7021 advanced non-small cell lung cancer patients who received chemotherapy (three cohorts), immunotherapy (two cohorts), and chemoimmunotherapy (two cohorts) from five data sources, including a de-identified nationwide (US-based) NSCLC clinico-genomic database and two randomized, double-blind, phase 3 clinical trials. BMI was categorized as underweight, normal weight, overweight, or obese. Underweight patients were excluded because of their small proportion. The primary endpoints were the associations between BMI and progression-free survival (PFS) and overall survival (OS) stratified by treatment type and sex, which were assessed using Kaplan–Meier methods and adjusted Cox modeling. Meta-analyses were performed to combine the adjusted hazard ratios.

**Results:**

In the pooled analysis, obesity was significantly associated with improved OS in patients receiving chemotherapy (hazard ratios [HR] = 0.84, 95% confidence interval (CI) 0.76–0.93), but there was no association with PFS (HR = 0.91, 95% CI 0.82–1.02). The association of BMI with OS for patients receiving chemotherapy differed by sex, with an inverse association in men (HR = 0.74, 95% CI 0.64–0.84), but no association observed in women (HR = 0.96, 95% CI 0.81–1.13, P_interactio*n* =_ 0.018). No impact of BMI on OS or PFS was detected in patients receiving immunotherapy or chemoimmunotherapy. Obese patients had the lowest level of tumor mutational burden, similar level of programmed death-ligand 1 expression and ESTIMATE scores.

**Conclusions:**

Obesity may be associated with an increased overall survival among male patients treated with chemotherapy, whereas not associated with the outcomes in patients treated with immunotherapy or chemoimmunotherapy.

**Supplementary Information:**

The online version contains supplementary material available at 10.1186/s12916-024-03688-2.

## Background

Advanced non-small cell lung cancer (NSCLC) is an aggressive disease that is associated with a poor prognosis. However, with the use of immune checkpoint inhibitors (ICIs), the prognosis of this disease has improved significantly. Despite the availability of new therapeutic agents and regimens, the overall survival (OS) of patients with NSCLC remains heterogeneous. Therefore, a better understanding of the clinical factors affecting patient outcomes may help to improve individualized treatment.

Obesity is a growing epidemic worldwide and is commonly diagnosed using the body mass index (BMI) metric, which is determined by dividing a person’s weight in kilograms by the square of their height in meters [1]. In a pooled analysis including 16 articles with more than 25,000 patients, Shepshelovich et al. found that NSCLC patients with high BMI showed better OS [2]. Similarly, in a pooled analysis including 63 lung cancer trials (*n* = 10,128), Oswalt et al. suggested that BMI > 28 was associated with longer OS in patients with NSCLC [3]. In a recent large meta-analysis including 203 studies with 6,320,365 participants, the authors indicated that patients with obesity and lung cancer had a lower risk of death than did those without obesity [4].

However, the association of BMI with survival outcomes in patients with advanced NSCLC treated with chemotherapy, immunotherapy, or chemoimmunotherapy as first-line therapy remains controversial. For example, an Italian study (*n* = 962) found that baseline obesity was associated with significantly improved outcomes in advanced NSCLC patients receiving first-line ICIs treatment [5]. However, the result was not verified in a Japanese study (*n* = 513) [6]. A pooled study including 2585 patients found that obese NSCLC patients responded better to first-line chemotherapy [7], but a more recent study (Italy, *n* = 426) did not confirm the result [5]. In addition, a recent international multicenter study (*n* = 853) found no association between baseline BMI and outcomes in advanced NSCLC patients receiving chemoimmunotherapy [8], but no further study has confirmed this finding. Although programmed death-ligand 1 (PD-L1) expression and tumor mutational burden (TMB) have been approved as clinical biomarkers of response to ICIs, the assessment is still challenging, due to inadequate sample, spatial and temporal heterogeneity [9]. In addition, they have been proven inadequate in accurately predicting treatment outcomes in the setting of chemoimmunotherapy [10]. Furthermore, the biomarker for chemotherapy efficacy in NSCLC is still lacking. Consequently, it is important to identify clinical characteristics that are predictive of treatment outcomes.

Accordingly, we aimed to investigate the association of BMI with progression-free survival (PFS) and OS in independent cohorts of advanced NSCLC patients treated with chemotherapy, immunotherapy, and chemoimmunotherapy as first-line therapy. Because of the disparity in outcomes for NSCLC between sexes, as well as sex differences in body composition [11], we assessed associations in male and female patients individually.

## Methods

### Study design and patient population

This was a retrospective multicenter cohort study. The analysis set included patients who met the following criteria: (1) NSCLC confirmed by histology; (2) received at least one dose of treatment; (3) had pretreatment baseline body weight and height; and (4) had available survival data.

The data come from seven cohorts of advanced NSCLC patients treated with three categories of therapies as first-line treatment: chemotherapy combined with or without bevacizumab (three cohorts), immunotherapy (two cohorts), and chemoimmunotherapy (two cohorts). Three cohorts (one category of therapy each) used the nationwide (US-based) de-identified Flatiron Health-Foundation Medicine NSCLC clinico-genomic database (FH-FMI CGDB). The de-identified data originated from approximately 280 US cancer clinics (~ 800 sites of care). Retrospective longitudinal clinical data were derived from electronic health record data, comprising patient-level structured and unstructured data, curated via technology-enabled abstraction, and were linked to genomic data derived from FMI comprehensive genomic profiling (CGP) tests in the FH-FMI CGDB by de-identified, deterministic matching [12]. Genomic alterations were identified via CGP of > 300 cancer-related genes on FMI’s next-generation sequencing (NGS) test [13–15]. The study included 5588 patients diagnosed with NSCLC from 2003 to 2021 who underwent FMI CGP testing.

In the CGDB-Chemo cohort (*n* = 2246), the QL1101 cohort (*n* = 502) and the SHChest-Chemo cohort (*n* = 443), we investigated the association between baseline BMI and clinical outcomes in patients who were treated with chemotherapy, with or without bevacizumab [16, 17]. We also assessed the association in advanced NSCLC treated with immunotherapy in the CGDB-Immu cohort (*n* = 1386) and the Chowell-Immu cohort (*n* = 172) [18]. The CGDB-Chemoimmu cohort (*n* = 1956) and the AK105-302 cohort (*n* = 316) were used to examine whether BMI was associated with survival outcomes in patients with advanced NSCLC treated with chemoimmunotherapy [19]. The full list of eligibility criteria for the patients included in these cohorts is included in the Additional file 1: eligibility criteria [16–19].

To gain insight into the potential mechanisms underlying the effect of obesity on survival, we assessed the association between BMI and ESTIMATE (Estimation of STromal and Immune cells in MAlignant Tumour tissues using Expression data) score, based on a published study [20].

The study was conducted by the Declaration of Helsinki and relevant guidelines/regulations published by the European Medicines Agency’s Committee. The ethical documents and study protocol were issued and approved by the Ethics Committees of Shanghai Chest Hospital (Reference number: IS2118). The clinical data of patients enrolled were all processed under anonymized conditions to fully ensure patient privacy. The ethics committees waived written informed consent because this was a retrospective study.

### Anthropometric measurements

For CGDB based cohorts and Chowell-Immu cohort, BMI was categorized according to World Health Organization (WHO) criteria: underweight (< 18.5), normal weight (18.5–24.9), overweight (25–29.9), or obese (≥ 30) [21]. Asian populations, including Chinese, have an increased risk of diabetes and cardiovascular diseases, even at a BMI < 25 [22]. Thus, in the remaining cohorts, BMI was categorized according to WHO Asia–Pacific region criteria: underweight (< 18.5), normal weight (18.5–22.9), overweight (23- 24.9), or obese (≥ 25) [23]. Underweight patients, comprising a small proportion of these cohorts (less than 5%), were excluded.

### Outcomes

The primary endpoints were PFS and OS stratified by cohort and sex. PFS was defined as the time from the start of the therapy to the date of disease progression or death, whichever occurred first. OS was defined as the time from the start of therapy to death from any cause. The secondary outcomes included objective response rate (ORR) and adverse events (AEs). Disease progression and response were assessed using Response Evaluation Criteria in Solid Tumors (RECIST) 1.1 criteria by radiologists or clinicians at each institution. The AEs were graded according to the National Cancer Institute (NCI) Common Terminology Criteria for Adverse Events (CTCAE) v5.0.

### Statistical analysis

Between-group differences were evaluated using the Chi-square test and Fisher’s exact test for categorical variables, and the Mann–Whitney U test and Kruskal–Wallis test for continuous variables, as appropriate.

In each individual clinical cohort, the associations of baseline BMI category with PFS and OS were assessed. Survival analysis was performed by the Kaplan–Meier method. Univariate and multivariate Cox proportional-hazards models were utilized to estimate the hazard ratio (HR) and 95% confidence interval (CI) for the outcome. Logistic regression was used for the analysis of ORR.

We conducted a meta-analysis using a random effects model and combined adjusted HRs to investigate the role of baseline BMI on survival in all cohorts, by therapy type and sex. Whether the association between BMI group and survival differed between men and women was determined by an interaction term in the Cox proportional hazards regression model.

Associations between risk of outcome and BMI on a continuous scale were examined using restricted cubic spline (RCS) analysis, with knots at equally spaced percentiles [24].

For all analyses, statistical significance was set at P values of less than 0.05. The statistical analyses were performed using R version 3.6.1 (R Project for Statistical Computing) and SPSS version 23.0 (IBM, Armonk, NY).

The data analysis in FH-FMI CGDB database is separately conducted by Shanghai Roche Pharmaceuticals Ltd. and we only have the report available for the FH-FMI CGDB cohorts reported in this study.

## Results

In the seven cohorts, a total of 7021 patients with advanced NSCLC were treated with chemotherapy, immunotherapy, or chemoimmunotherapy between 2003 and 2021. Most patients (*n* = 6203) were enrolled from real-life settings and 818 patients were enrolled from two phase 3 clinical trials [16, 19]. Table 1 summarizes the patient characteristics of the seven cohorts.

**Table 1:** Patient characteristics in each cohort

	**CGDB-Chemo cohort (*n* = 2246)**			**CGDB-Immu cohort (*n* = 1386)**			**CGDB-Chemoimmu cohort (*n* = 1956)**			**QL1101 cohort (*n* = 502)**		
	**Normal weight**	**Overweight**	**Obese**	**Normal weight**	**Overweight**	**Obese**	**Normal weight**	**Overweight**	**Obese**	**Normal weight**	**Overweight**	**Obese**
**No. of patients**	902 (40.2)	770 (34.3)	574 (25.5)	618 (44.6)	470 (33.9)	298 (21.5)	836 (42.7)	658 (33.7)	462 (23.6)	261 (52.0)	116 (23.1)	125 (24.9)
**Median age (IQR)**	74 (66–80)	75 (67–81)	73 (66–79.8)	76 (68–81)	76.5 (68–83)	73 (66–79)	72 (64–78)	72 (65–78)	71.5 (65–76)	59 (52–64)	57.5 (49–65)	58 (50–64)
**Gender**												
Female	434 (48.1)	308 (40.0)	273 (47.6)	337 (54.5)	214 (45.5)	144 (48.3)	383 (45.8)	265 (40.3)	204 (44.2)	97 (37.2)	57 (49.1)	49 (39.2)
Male	468 (51.9)	462 (60.0)	301 (52.4)	281 (45.5)	256 (54.5)	154 (51.7)	453 (51.2)	393 (59.7)	258 (55.8)	164 (62.8)	59 (50.9)	76 (60.8)
**ECOG PS**												
0	275 (30.5)	287 (37.3)	189 (32.9)	162 (26.2)	136 (28.9)	76 (25.5)	306 (36.6)	249 (37.8)	168 (36.4)	44 (16.9)	33 (28.4)	44 (35.2)
1	453 (50.2)	357 (46.4)	270 (47.0)	299 (48.4)	229 (48.7)	157 (52.7)	384 (46.0)	298 (45.3)	210 (45.4)	217 (83.1)	83 (71.6)	81 (64.8)
> = 2	174 (19.3)	126 (16.3)	115 (20.1)	157 (25.4)	105 (22.4)	65 (21.8)	146 (17.4)	111 (16.9)	84 (18.2)	0 (0)	0 (0)	0 (0)
**Smoking status**												
History of smoking	811 (89.9)	671 (87.1)	505 (88.0)	577 (93.4)	433 (92.1)	276 (92.6)	766 (91.6)	597 (90.7)	411 (89.0)	139 (53.3)	48 (41.4)	53 (42.4)
No history of smoking	90 (10.0)	97 (12.6)	68 (11.9)	41 (6.6)	36 (7.7)	22 (7.4)	70 (8.4)	61 (9.3)	51 (11.0)	122 (46.7)	68 (58.6)	72 (57.6)
Missing	1 (0.1)	2 (0.3)	1 (0.1)	0 (0)	1 (0.2)	0 (0)	0 (0)	0 (0)	0 (0)	0 (0)	0 (0)	0 (0)
**Type of tumor**												
Non-squamous	584 (64.8)	507 (65.8)	377 (65.7)	413 (66.8)	328 (69.8)	202 (67.8)	610 (73.0)	486 (73.9)	326 (70.6)	261 (100)	116 (100)	125 (100)
Squamous	270 (29.9)	235 (30.6)	181 (31.5)	175 (28.3)	126 (26.8)	85 (28.5)	186 (22.3)	148 (22.4)	117 (25.3)	0 (0)	0 (0)	0 (0)
Other	48 (5.3)	28 (3.6)	16 (2.8)	30 (4.9)	16 (3.4)	11 (3.7)	40 (4.7)	24 (3.7)	19 (4.1)	0 (0)	0 (0)	0 (0)
**Stage**												
III	372 (41.2)	361 (46.9)	261 (45.4)	198 (32.0)	162 (34.5)	118 (39.6)	258 (30.9)	219 (33.3)	174 (37.7)	8 (3.1)	4 (3.4)	8 (6.4)
IV	530 (58.8)	409 (53.1)	313 (54.5)	420 (68.0)	308 (65.5)	180 (60.4)	578 (69.1)	439 (66.7)	288 (62.3)	253 (96.9)	112 (96.6)	117 (93.6)
**Brain metastases**												
No	627 (69.5)	562 (73.0)	418 (72.8)	461 (74.6)	345 (73.4)	227 (76.2)	600 (71.8)	494 (75.1)	356 (77.1)	240 (92.0)	114 (98.3)	118 (94.4)
Yes	275 (30.5)	208 (27.0)	156 (27.2)	157 (25.4)	125 (26.6)	71 (23.8)	236 (28.2)	164 (24.9)	106 (22.9)	21 (8.0)	2 (1.7)	7 (5.6)
**Bone metastases**												
No	531 (58.9)	464 (60.3)	369 (64.3)	363 (58.7)	291 (61.9)	188 (63.1)	459 (54.9)	384 (58.4)	277 (60.0)	258 (98.9)	116 (100.0)	124 (100.0)
Yes	371 (41.1)	306 (39.7)	205 (35.7)	255 (41.3)	179 (38.1)	110 (36.9)	377 (45.1)	274 (41.6)	185 (40.0)	3 (1.1)	0 (0)	0 (0)
**Liver metastases**												
No	697 (77.3)	622 (80.8)	453 (78.9)	508 (82.2)	384 (81.7)	235 (78.9)	694 (83.0)	532 (80.9)	379 (82.0)	232 (88.9)	110 (94.8)	120 (96.0)
Yes	205 (22.7)	148 (19.2)	121 (21.1)	110 (17.8)	86 (18.3)	63 (21.1)	142 (17.0)	126 (19.1)	83 (18.0)	29 (11.1)	6 (5.2)	5 (4.0)
**PD-L1 expression**												
< 1%	42 (4.7)	17 (2.1)	22 (3.8)	15 (2.4)	17 (3.6)	9 (3.1)	36 (4.3)	43 (6.5)	20 (4.3)	–	–	–
1% < = PDL1 < 50%	102 (11.3)	76 (9.9)	59 (10.3)	64 (10.4)	49 (10.4)	43 (14.4)	126 (15.1)	76 (11.5)	73 (15.8)	–	–	–
> = 50%	43 (4.8)	39 (5.1)	25 (4.4)	155 (25.1)	100 (21.3)	65 (21.8)	70 (8.4)	53 (8.1)	36 (7.8)	–	–	–
Missing	715 (79.2)	638 (82.9)	468 (81.5)	384 (62.1)	304 (64.7)	181 (60.7)	604 (72.2)	486 (73.9)	333 (72.1)	–	–	–
EGFR** mutation**												
Negative	351 (38.9)	308 (40.0)	199 (34.7)	249 (40.3)	168 (35.7)	126 (42.3)	282 (33.7)	234 (35.6)	166 (35.9)	157 (60.2)	64 (55.2)	84 (67.2)
Positive	18 (2.0)	21 (2.7)	14 (2.4)	5 (0.8)	5 (1.1)	6 (2.0)	7 (0.8)	12 (1.8)	4 (0.9)	104 (39.8)	52 (44.8)	41 (32.8)
Missing	533 (59.1)	441 (57.3)	361 (62.9)	364 (58.9)	297 (63.2)	166 (55.7)	547 (65.5)	412 (62.6)	292 (63.2)	0 (0)	0 (0)	0 (0)

	**SHChest-Chemo cohort (*n* = 443)**			**Chowell-Immu cohort (*n* = 172)**			**AK105-302 cohort (*n* = 316)**		
	**Normal weight**	**Overweight**	**Obese**	**Normal weight**	**Overweight**	**Obese**	**Normal weight**	**Overweight**	**Obese**
**No. of patients**	270 (60.9)	76 (17.2)	97 (21.9)	62 (36.0)	62 (36.0)	48(28.0)	146 (46.2)	84 (26.6)	86 (27.2)
**Median age (IQR)**	59 (53–65)	62 (54.5–66)	55 (50–62)	65.4 (59.5–74.4)	64.3 (58.3–72.3)	67.6 (60.8–72.0)	62 (57–66)	60 (55–65)	61 (55–66)
**Gender**									
Female	84 (31.1)	13 (17.1)	42 (56.7)	39 (62.9)	25 (40.3)	19 (39.6)	7 (4.8)	11 (13.1)	7 (8.1)
Male	186 (68.9)	63 (82.9)	55 (56.7)	23 (37.1)	37 (59.7)	29 (60.4)	139 (95.2)	73 (86.9)	79 (91.9)
**ECOG PS**									
0	104 (38.6)	32 (42.1)	32 (33.0)	18 (29.0)	25 (40.3)	22 (45.8)	33 (22.6)	21 (25.0)	20 (23.3)
1	110 (40.7)	27 (35.5)	49 (50.5)	41 (66.1)	34 (54.8)	24 (50.0)	113 (77.4)	63 (75.0)	66 (76.7)
> = 2	56 (20.7)	17 (22.4)	16 (16.5)	3 (4.8)	3 (4.8)	2 (4.2)	0 (0)	0 (0)	0 (0)
**Smoking status**									
History of smoking	140 (51.9)	49 (64.5)	32 (33.0)	–	–	–	131 (89.7)	71 (84.5)	75 (87.2)
No history of smoking	130 (48.1)	27 (35.5)	65 (67.0)	–	–	–	15 (10.3)	13 (15.5)	11 (12.8)
Missing	0 (0)	0 (0)	0 (0)	–	–	–	0 (0)	0 (0)	0 (0)
**Type of tumor**									
Non-squamous	186 (68.9)	50 (65.8)	77 (79.4)	–	–	–	0 (0)	0 (0)	0 (0)
Squamous	84 (31.1)	26 (34.2)	20 (20.6)	–	–	–	146 (100)	84 (100)	86 (100)
Other	0 (0)	0 (0)	0 (0)	–	–	–	0 (0)	0 (0)	0 (0)
**Stage**									
III	97 (35.9)	23 (30.3)	33 (34.0)	2 (3.2)	0 (0)	0 (0)	18 (12.3)	13 (15.5)	12 (14.0)
IV	173 (64.1)	53 (69.7)	64 (66.0)	60 (96.8)	62 (100)	48 (100)	128 (87.7)	71 (84.5)	74 (86.0)
**Brain metastases**									
No	–	–	–	–	–	–	140 (95.9)	83 (98.8)	84 (97.7)
Yes	–	–	–	–	–	–	6 (4.1)	1 (1.2)	2 (2.3)
**Bone metastases**									
No	–	–	–	–	–	–	114 (78.1)	65 (77.4)	65 (75.6)
Yes	–	–	–	–	–	–	32 (21.9)	19 (22.6)	21 (24.4)
**Liver metastases**									
No	–	–	–	–	–	–	125 (85.6)	73 (86.9)	79 (91.9)
Yes	–	–	–	–	–	–	21 (14.4)	11 (13.1)	7 (8.1)
**PD-L1 expression**									
< 1%	–	–	–	–	–	–	50 (34.2)	28 (33.7)	28 (32.6)
1% < = PDL1 < 50%	–	–	–	–	–	–	72 (49.3)	39 (47.0)	39 (45.3)
> = 50%	–	–	–	–	–	–	24 (16.4)	16 (19.3)	19 (22.1)
Missing	–	–	–	–	–	–	0 (0)	0 (0)	0 (0)
EGFR** mutation**									
Negative	226 (83.7)	65 (85.5)	67 (69.1)	–	–	–	146 (100)	84 (100)	86 (100)
Positive	44 (16.3)	11 (14.5)	30 (30.9)	–	–	–	0 (0)	0 (0)	0 (0)
Missing	0 (0)	0 (0)	0 (0)	–	–	–	0 (0)	0 (0)	0 (0)

Data are n (%). – = not available

*IQR* Interquartile range, *ECOG PS* Eastern cooperative oncology group performance status, *PD-L1* Programmed death-ligand 1, *EGFR* Epidermal growth factor receptor

**Fig. 1:**
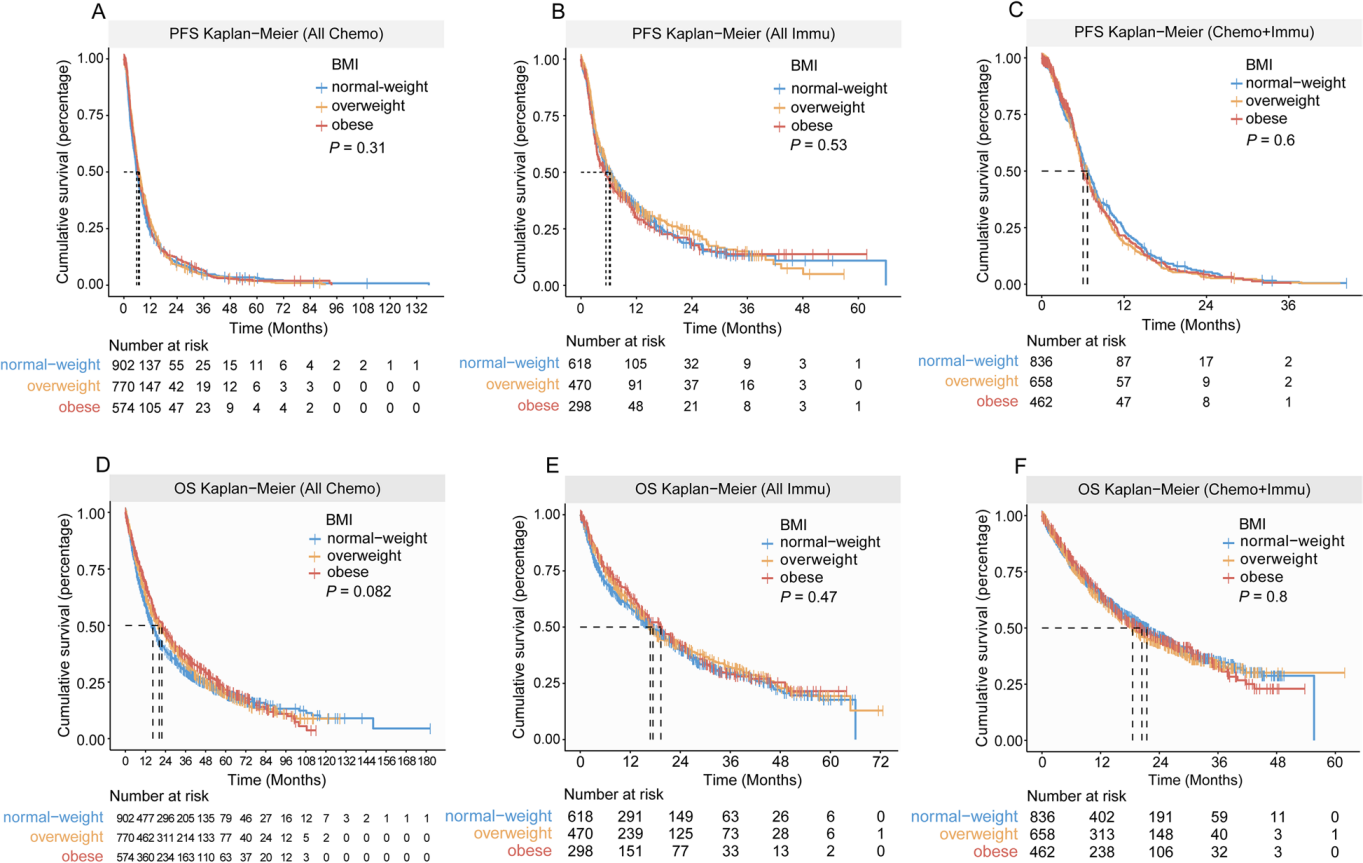
Progression-free survival and overall survival by BMI category. Progression-free survival in patients in the **A** CGDB-Chemo cohort, **B** CGDB-Immu cohort, **C** CGDB-Chemoimmu cohort. Overall survival in patients in the **D** CGDB-Chemo cohort, **E** CGDB-Immu cohort, **F** CGDB-Chemoimmu cohort

The detailed baseline characteristics of CGDB-Chemo cohort are shown in Additional file 1: Table S1. In this cohort, no significant association between BMI and PFS was found in either the univariable or multivariable analyses (Fig. 1A, Table 2). However, obese patients showed improved OS compared with patients with normal weight in the univariable and multivariable analyses (Fig. 1D, Table 3). The restricted cubic spline model showed a linear association between BMI and HR for OS (P _non-linearity_ = 0.171; Additional file 1: Fig. S1).

**Table 2:** Association between BMI and progression-free survival

	**Events/patients**	**Median, months (95% CI)**	**Univariable**		**Multivariable**	
			**HR (95% CI)**	***p*** **value**	**HR (95% CI)**	*p* **value**
**CGDB-Chemo cohort**^**a**^						
All patients (*n* = 2246)						
Normal weight	638/902	5.75 (5.42–6.60)	1 (ref)		1 (ref)	
Overweight	560/770	6.97 (6.18–7.69)	0.93 (0.83–1.04)	0.177	0.93 (0.83–1.05)	0.225
Obese	435/574	6.60 (5.95–7.16)	0.93 (0.82–1.05)	0.212	0.93 (0.83–1.05)	0.265
Men (*n* = 1231)						
Normal weight	318/468	5.42 (5.00–5.78)	1 (ref)		1 (ref)	
Overweight	323/462	6.41 (5.62–7.82)	0.86 (0.74–1.09)	0.064	0.84 (0.72–0.99)	0.037
Obese	232/301	6.05 (5.22–7.13)	0.97 (0.82–1.15)	0.729	0.96 (0.81–1.14)	0.624
Women (*n* = 1015)						
Normal weight	320/434	6.77 (5.59–7.79)	1 (ref)		1 (ref)	
Overweight	237/308	7.16 (6.37–8.25)	0.97 (0.82–1.14)	0.682	0.97 (0.82–1.15)	0.710
Obese	203/273	6.90 (6.14–8.74)	0.88 (0.73–1.04)	0.137	0.88 (0.74–1.05)	0.146
**CGDB-Immu cohort**^**a**^						
All patients (*n* = 1386)						
Normal weight	331/618	6.14 (5.09–7.43)	1 (ref)		1 (ref)	
Overweight	276/470	6.34 (5.32–7.46)	0.95 (0.81–1.11)	0.507	0.97 (0.83–1.15)	0.751
Obese	184/298	5.39 (3.75–6.77)	1.05 (0.88–1.26)	0.569	1.01 (0.84–1.21)	0.932
Men (*n* = 691)						
Normal weight	151/281	6.14 (4.57–8.02)	1 (ref)		1 (ref)	
Overweight	148/256	6.54 (5.22–9.63)	0.90 (0.72–1.13)	0.377	0.96 (0.76–1.21)	0.730
Obese	91/154	5.52 (3.48–9.76)	1.03 (0.80–1.34)	0.810	0.98 (0.75–1.27)	0.857
Women (*n* = 695)						
Normal weight	180/337	6.41 (5.00–8.35)	1 (ref)		1 (ref)	
Overweight	128/214	6.14 (4.63–7.44)	1.00 (0.80–1.25)	0.993	1.01 (0.80–1.27)	0.963
Obese	93/144	5.09 (3.35–6.60)	1.10 (0.85–1.41)	0.477	1.05 (0.82–1.36)	0.700
**CGDB-Chemoimmu cohort**^**a**^						
All patients (*n* = 1956)						
Normal weight	445/836	6.64 (6.05–7.33)	1 (ref)		1 (ref)	
Overweight	374/658	6.67 (5.72–7.00)	1.07 (0.93–1.23)	0.337	1.07 (0.93–1.23)	0.321
Obese	261/462	6.01 (5.59–7.00)	1.05 (0.90–1.23)	0.518	1.07 (0.91–1.25)	0.405
Men (*n* = 1104)						
Normal weight	229/453	6.93 (5.91–7.62)	1 (ref)		1 (ref)	
Overweight	226/393	6.67 (5.72–7.03)	1.11 (0.93–1.34)	0.256	1.12 (0.93–1.35)	0.234
Obese	148/258	5.72 (5.36–7.06)	1.13 (0.92–1.40)	0.234	1.14 (0.92–1.42)	0.219
Women (*n* = 852)						
Normal weight	216/383	6.57 (5.85–7.49)	1 (ref)		1 (ref)	
Overweight	148/265	6.44 (5.16–7.66)	1.04 (0.84–1.28)	0.749	1.04 (0.84–1.28)	0.726
Obese	113/204	6.28 (5.78–7.49)	0.96 (0.76–1.21)	0.727	0.99 (0.79–1.25)	0.947
**QL1101 cohort**^**b**^						
All patients (*n* = 500)						
Normal weight	207/259	8.03 (7.30–8.77)	1 (ref)		1 (ref)	
Overweight	90/116	8.67 (7.87–9.47)	0.77 (0.60–0.99)	0.042	0.82 (0.63–1.05)	0.114
Obese	97/125	7.53 (7.09–9.98)	0.31 (0.60–0.98)	0.031	0.83 (0.64–1.06)	0.136
Men (*n* = 298)						
Normal weight	134/163	7.03 (6.10–7.97)	1 (ref)		1 (ref)	
Overweight	50/59	8.53 (8.06–9.00)	0.68 (0.49–0.94)	0.021	0.76 (0.54–1.06)	0.108
Obese	65/76	7.70 (6.53–8.87)	0.81 (0.60–1.10)	0.173	0.87 (0.63–1.20)	0.392
Women (*n* = 202)						
Normal weight	73/96	9.70 (8.50–10.90)	1 (ref)		1 (ref)	
Overweight	40/57	9.20 (7.05–11.35)	0.89 (0.61–1.32)	0.572	0.92 (0.62–1.36)	0.666
Obese	32/49	10.03 (8.89–11.17)	0.68 (0.45–1.04)	0.072	0.70 (0.46–1.08)	0.108
**Chowell-Immu cohort**^**c**^						
All patients (*n* = 172)						
Normal weight	44/62	3.38 (1.46–5.31)	1 (ref)		1 (ref)	
Overweight	49/62	5.29 (2.03–8.55)	1.02 (0.68–1.53)	0.933	1.33 (0.85–2.09)	0.215
Obese	37/48	4.90 (1.96–7.83)	1.05 (0.68–1.63)	0.815	1.14 (0.68–1.92)	0.610
Men (*n* = 89)						
Normal weight	13/23	3.45 (0.00–15.62)	1 (ref)		1 (ref)	
Overweight	32/37	3.94 (1.87–6.02)	1.75 (0.91–3.35)	0.092	2.14 (1.02–4.51)	0.045
Obese	23/29	4.37 (0.10–8.66)	1.67 (0.84–3.31)	0.141	1.23 (0.53–2.83)	0.628
Women (*n* = 83)						
Normal weight	31/39	3.38 (0.65–6.12)	1 (ref)		1 (ref)	
Overweight	17/25	8.02 (4.72–11.31)	0.60 (0.33–1.09)	0.092	0.55 (0.27–1.13)	0.102
Obese	14/19	6.21 (2.47–9.95)	0.71 (0.38–1.34)	0.289	0.77 (0.37–1.61)	0.771

^a^Adjusted for age, gender, race, ECOG PS, type of tumor, smoking status, stage, site of metastases, genetic mutations, tissue tumor mutational burden, and PD-L1 expression

^b^Adjusted for age, gender, ECOG PS, smoking status, stage, site of metastases, and *EGFR* mutation

^c^Adjusted for age, gender, stage, ECOG PS, loss of heterozygosity status, neutrophil-to-lymphocyte ratio, hemoglobin, platelets, tumor mutational burden, fraction of copy number alteration, HLA-I evolutionary divergence, and treatment type

**Table 3:** Association between BMI and overall survival

	**Events/patients**	**Median, months (95% CI)**	**Univariable**		**Multivariable**	
			**HR (95% CI)**	***p* value**	**HR (95% CI)**	***p* value**
**CGDB-Chemo cohort**^**a**^						
All patients (*n* = 2246)						
Normal weight	652/902	16.43 (14.26–18.23)	1 (ref)		1 (ref)	
Overweight	578/770	20.21 (17.58–23.33)	0.93 (0.83–1.01)	0.281	0.93 (0.83–1.05)	0.232
Obese	411/574	21.82 (18.30–25.73)	0.87 (0.77–0.99)	0.028	0.87 (0.77–0.98)	0.027
Men (*n* = 1231)						
Normal weight	357/468	12.12 (10.58–14.92)	1 (ref)		1 (ref)	
Overweight	353/462	18.04 (14.52–22.44)	0.80 (0.69–0.93)	0.003	0.82 (0.71–0.96)	0.011
Obese	217/301	18.17 (15.67–22.34)	0.76 (064–0.90)	0.001	0.77 (0.65–0.92)	0.003
Women (*n* = 1015)						
Normal weight	295/434	20.11 (18.00–26.61)	1 (ref)		1 (ref)	
Overweight	225/308	22.51 (18.43–29.47)	1.05 (0.88–1.25)	0.582	1.04 (0.881.24)	0.621
Obese	194/273	26.55 (21.72–32.53)	0.99 (0.83–1.19)	0.940	0.98 (0.82–1.18)	0.848
**CGDB-Immu cohort**^**a**^						
All patients (*n* = 1386)						
Normal weight	366/618	17.45 (14.23–20.34)	1 (ref)		1 (ref)	
Overweight	282/470	16.79 (15.34–18.99)	0.93 (0.79–1.08)	0.334	0.94 (0.80–1.10)	0.429
Obese	170/298	19.35 (15.38–23.33)	0.91 (0.76–1.09)	0.287	0.91 (0.76–1.09)	0.309
Men (*n* = 691)						
Normal weight	173/281	15.51 (12.32–18.86)	1 (ref)		1 (ref)	
Overweight	161/256	15.80 (12.75–18.27)	0.93 (0.75–1.15)	0.505	0.96 (0.77–1.19)	0.685
Obese	93/154	16.20 (13.40–22.14)	0.97 (0.75–1.25)	0.807	0.97 (0.75–1.25)	0.797
Women (*n* = 695)						
Normal weight	193/337	20.30 (14.36–23.03)	1 (ref)		1 (ref)	
Overweight	121/214	18.86 (16.07–26.84)	0.91 (0.72–1.14)	0.389	0.93 (0.74–1.17)	0.517
Obese	77/144	21.59 (16.26–16.48)	0.84 (0.64–1.09)	0.193	0.84 (0.65–1.10)	0.206
**CGDB-Chemoimmu cohort**^**a**^						
All patients (*n* = 1956)						
Normal weight	405/836	21.45 (18.50–24.25)	1 (ref)		1 (ref)	
Overweight	329/658	18.53 (16.30–21.22)	1.05 (0.91–1.21)	0.515	1.07 (0.92–1.23)	0.395
Obese	233/462	20.44 (16.49–24.38)	1.01 (0.86–1.19)	0.887	1.02 (0.87–1.20)	0.809
Men (*n* = 1104)						
Normal weight	234/453	19.31 (15.34–23.29)	1 (ref)		1 (ref)	
Overweight	211/393	16.99 (14.42–19.84)	1.02 (0.84–1.22)	0.864	1.03 (0.86–1.24)	0.739
Obese	145/258	16.30 (13.21–20.53)	1.06 (0.86–1.30)	0.594	1.07 (0.87–1.32)	0.530
Women (*n* = 852)						
Normal weight	171/383	23.42 (19.94–30.19)	1 (ref)		1 (ref)	
Overweight	118/265	20.27 (18.17–28.42)	1.06 (0.84–1.34)	0.641	1.08 (0.85–1.37)	0.514
Obese	88/204	30.32 (20.44–35.48)	0.93 (0.72–1.21)	0.601	0.94 (0.72–1.21)	0.620
**QL1101 cohort**^**b**^						
All patients (*n* = 500)						
Normal weight	152/259	19.30 (16.33–22.27)	1 (ref)		1 (ref)	
Overweight	57/116	23.27 (15.96–30.57)	0.75 (0.55–1.02)	0.065	0.88 (0.64–1.20)	0.410
Obese	62/125	26.23 (22.29–30.18)	0.72 (0.54–0.97)	0.029	0.73 (0.54–0.99)	0.045
Men (*n* = 298)						
Normal weight	108/163	14.40 (12.65–16.15)	1 (ref)		1 (ref)	
Overweight	35/59	16.43 (14.06–18.80)	0.77 (0.52–1.12)	0.175	0.77 (0.53–1.13)	0.179
Obese	39/76	26.23 (20.57–31.90)	0.60 (0.41–0.86)	0.006	0.54 (0.37–0.78)	0.001
Women (*n* = 202)						
Normal weight	44/96	26.90 (–)	1 (ref)		1 (ref)	
Overweight	22/57	–	0.85 (0.51–1.41)	0.523	0.93 (0.55–1.57)	0.791
Obese	23/49	–	1.03 (0.62–1.71)	0.909	1.11 (0.66–1.87)	0.687
**SHChest-Chemo cohort**^**c**^						
All patients (*n* = 443)						
Normal weight	201/270	17.0 (15.1–19.0)	1 (ref)		1 (ref)	
Overweight	66/76	10.4 (8.0–12.7)	1.61 (1.22–2.13)	0.001	1.53 (1.16–2.03)	0.003
Obese	67/97	19.7 (13.3–26.0)	0.73 (0.55–0.96)	0.025	0.78 (0.59–1.03)	0.076
Men (*n* = 304)						
Normal weight	143/186	15.6 (13.1–18.0)	1 (ref)		1 (ref)	
Overweight	54/63	11.0 (8.0–14.0)	1.45 (1.06–1.99)	0.020	1.45 (1.06–1.99)	0.021
Obese	42/55	18.8 (16.3–21.4)	0.72 (0.50–1.02)	0.067	0.79 (0.55–1.13)	0.203
Women (*n* = 139)						
Normal weight	58/84	20.0 (17.0–23.0)	1 (ref)		1 (ref)	
Overweight	12/13	10.0 (7.4–12.6)	1.96 (1.04–3.70)	0.037	2.16 (1.14–4.12)	0.019
Obese	25/42	28.8 (17.2–40.4)	0.76 (0.48–1.22)	0.259	0.68 (0.41–1.13)	0.133
**Chowell-Immu cohort**^**d**^						
All patients (*n* = 172)						
Normal weight	31/62	25. 50 (9.22–41.77)	1 (ref)		1 (ref)	
Overweight	32/62	22.70 (15.59–29.82)	1.12 (0.68–1.84)	0.667	1.39 (0.81–2.39)	0.235
Obese	23/48	21.39 (17.32–25.46)	1.02 (0.59–1.75)	0.956	0.98 (0.51–1.86)	0.975
Men (*n* = 89)						
Normal weight	11/23	33.02 (4.31–61.73)	1 (ref)		1 (ref)	
Overweight	23/37	14.95 (6.22–23.68)	1.52 (0.73–3.15)	0.262	2.09 (0.90–4.83)	0.085
Obese	15/29	20.37 (11.30–29.44)	1.88 (0.53–2.60)	0.685	0.56 (0.21–1.51)	0.563
Women (*n* = 83)						
Normal weight	20/39	25.50 (12.79–38.30)	1 (ref)		1 (ref)	
Overweight	9/25	26.05 (19.40–32.71)	0.66 (0.30–1.46)	0.308	0.82 (0.35–1.92)	0.649
Obese	8/19	26.32 (18.40–34.23)	0.76 (0.33–1.74)	0.513	1.12 (0.44–2.87)	0.813

– = not available

^a^Adjusted for age, gender, race, ECOG PS, type of tumor, smoking status, stage, site of metastases, genetic mutations, tissue tumor mutational burden, and PD-L1 expression

^b^Adjusted for age, gender, ECOG PS, smoking status, stage, site of metastases, and *EGFR* mutation

^c^Adjusted for age, gender, type of tumor, smoking status, stage, ECOG PS, and *EGFR* mutation

^d^Adjusted for age, gender, stage, loss of heterozygosity status, neutrophil-to-lymphocyte ratio, hemoglobin, platelets, tumor mutational burden, fraction of copy number alteration, HLA-I evolutionary divergence, and treatment type

The baseline demographic and disease characteristics of the QL1101 cohort are shown in Additional file 1: Table S2. With a median follow-up of 26 months in the cohort treated with chemotherapy and bevacizumab, there were 271 deaths and 394 PFS events. BMI was not associated with PFS (Additional file 1: Fig. S2A, Table 2) or ORR (Additional file 1: Table S3). Efficacy analysis showed a mean tumor shrinkage of 27.1% in normal weight patients, 26.2% in overweight patients, and 26.1% in obese patients (Additional file 1: Fig. S3). There was no significant difference among the three groups (P = 0.947). However, obese patients had significantly longer OS than did patients with normal weight (Additional file 1: Fig. S2D, Table 3).

Additional file 1: Table S4 lists the detailed demographic and clinical characteristics of the SHChest-Chemo cohort. With a median follow-up of 31 months, 334 patients had died. The difference between the obese and normal weight groups was marginally significant for OS (Additional file 1: Fig. S4A, Table 3).

**Fig. 2:**
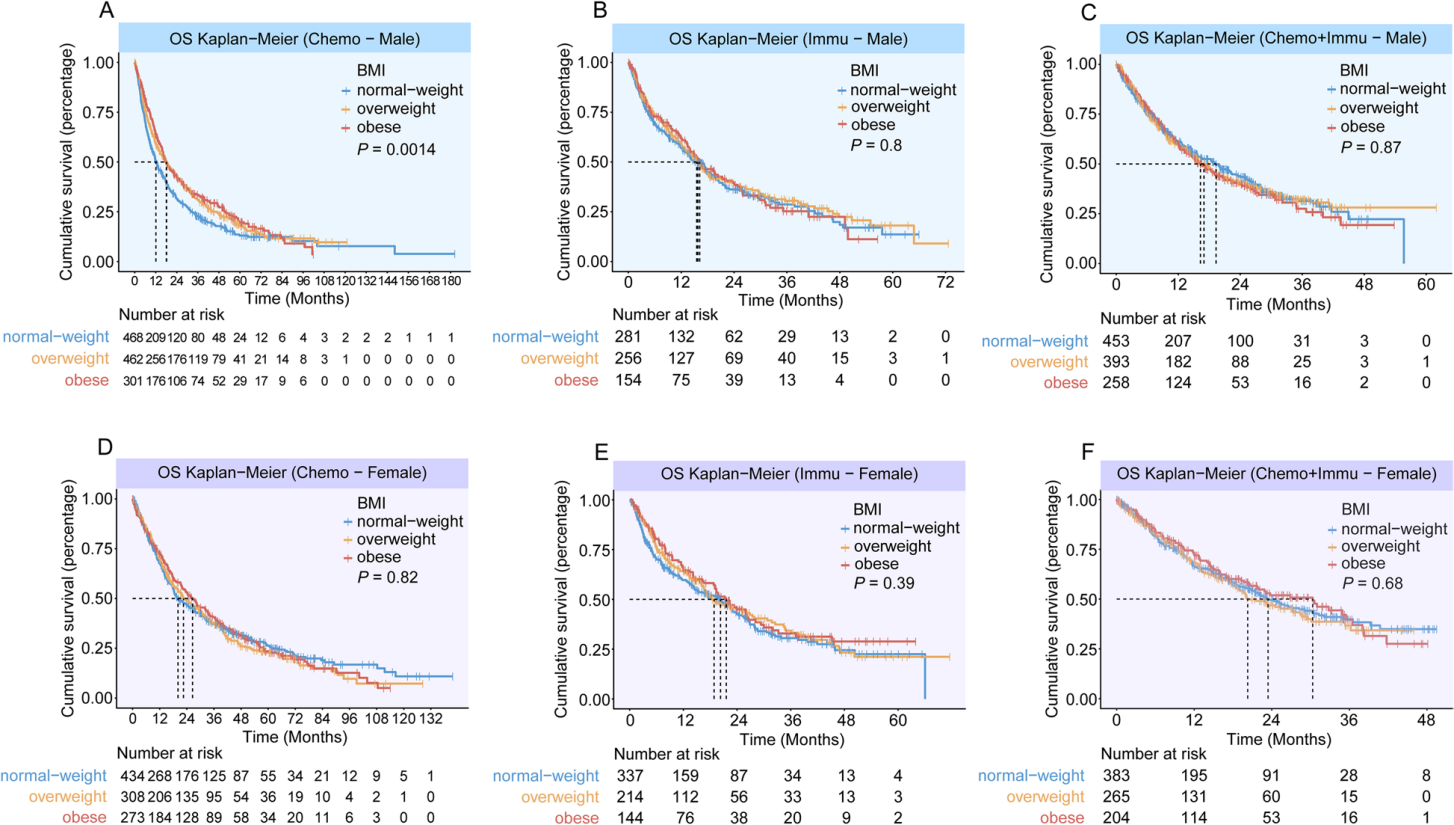
Overall survival by BMI category and sex. Overall survival in male patients in the **A** CGDB-Chemo cohort, **B** CGDB-Immu cohort, **C** CGDB-Chemoimmu cohort. Overall survival in female patients in the **D** CGDB-Chemo cohort, **E** CGDB-Immu cohort, **F** CGDB-Chemoimmu cohort

**Fig. 3:**
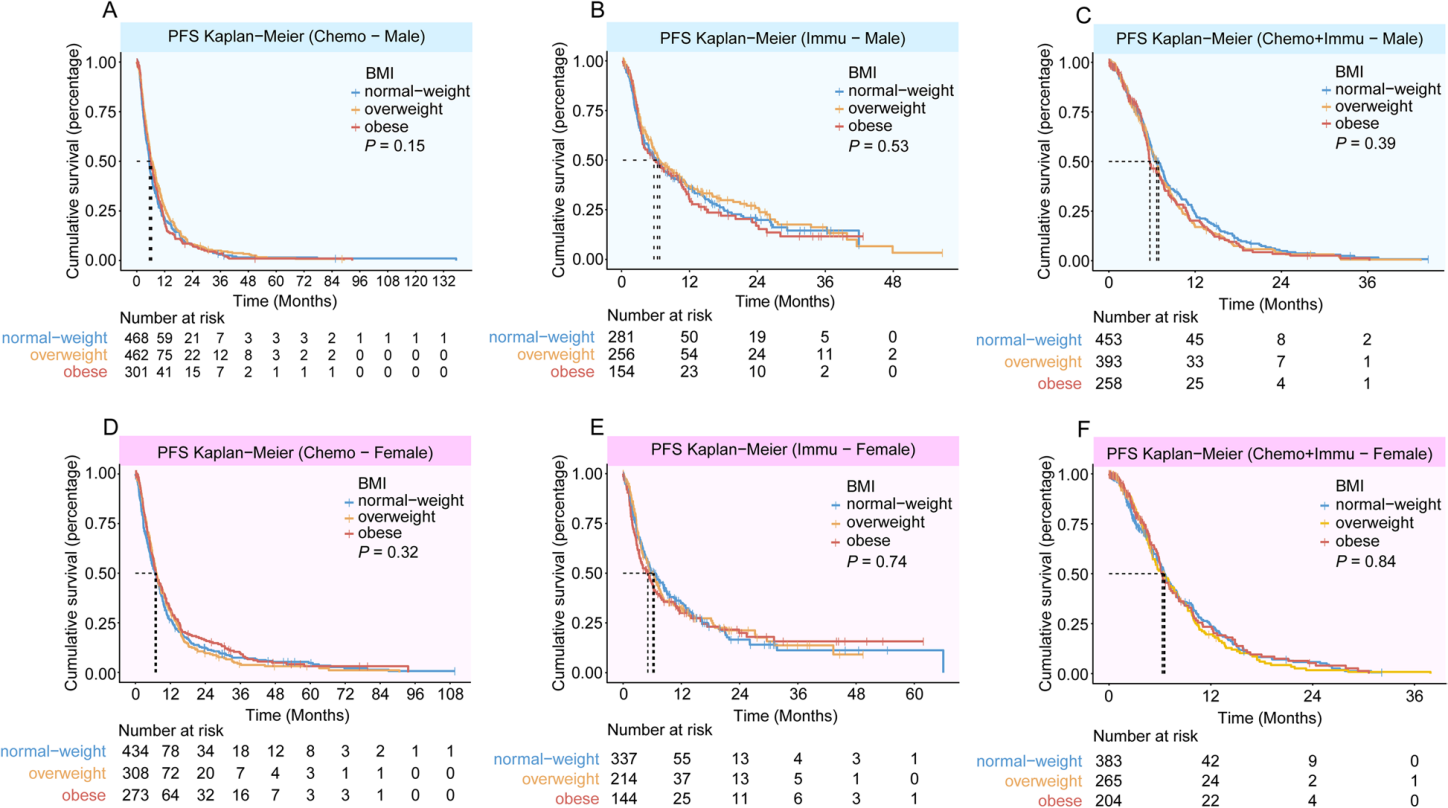
Progression-free survival by BMI category and sex. Progression-free survival in male patients in the **A** CGDB-Chemo cohort, **B** CGDB-Immu cohort, **C** CGDB-Chemoimmu cohort. Progression-free survival in female patients in the **D** CGDB-Chemo cohort, **E** CGDB-Immu cohort, **F** CGDB-Chemoimmu cohort

In male patients of the CGDB-Chemo cohort and QL1101 cohort, obesity was associated with improved OS compared with a normal BMI (Figs. 2A, Additional file 1: Fig. S2D, Table 3). In male patients of the SHChest-Chemo cohort, obesity was marginally significant for OS in the univariable analysis (Additional file 1: Fig. S4B, Table 3), but not in the multivariable analysis (Table 3). The PFS duration of the obese male patients did not differ from those of the patients with a normal BMI (Table 2). Additionally, PFS and OS did not differ significantly between obesity and normal weight in female patients (Figs. 2 and 3, Additional file 1: Fig. S2, S4 and Tables 2 and 3).

The characteristics of the two immunotherapy cohorts are shown in Additional file 1: Tables S5 and S6. In both univariable and multivariable analyses, obese patients were not observed to have a statistically significant PFS or OS benefit compared with those with a normal BMI, regardless of gender (Figs. 1–3, Additional file 1: Fig. S5 and Tables 2, 3). The ORR for immunotherapy according to BMI category, did not differ significantly, regardless of gender (Additional file 1: Table S3).

Additional file 1: Tables S7 and S8 summarizes patient characteristics of the two chemoimmunotherapy cohorts. In the CGDB-Chemoimmu cohort, no impact of BMI on PFS or OS was detected, regardless of gender (Figs. 1, 2, 3 and Tables 2, 3). Similar results were obtained in the AK105-302 cohort. A benefit with penpulimab plus chemotherapy with respect to PFS and OS was observed in BMI subgroups (Additional file 1: Fig. S6).

We evaluated the frequencies of AEs by BMI and grade in QL1101 and AK105-302 cohorts. The incidence of AEs did not significantly differ among the BMI categories (Additional file 1: Table S9). Similarly, there were no significant differences in the frequency of immune-related AEs across the BMI categories in the penpulimab plus chemotherapy arm (Additional file 1: Table S9).

**Fig. 4:**
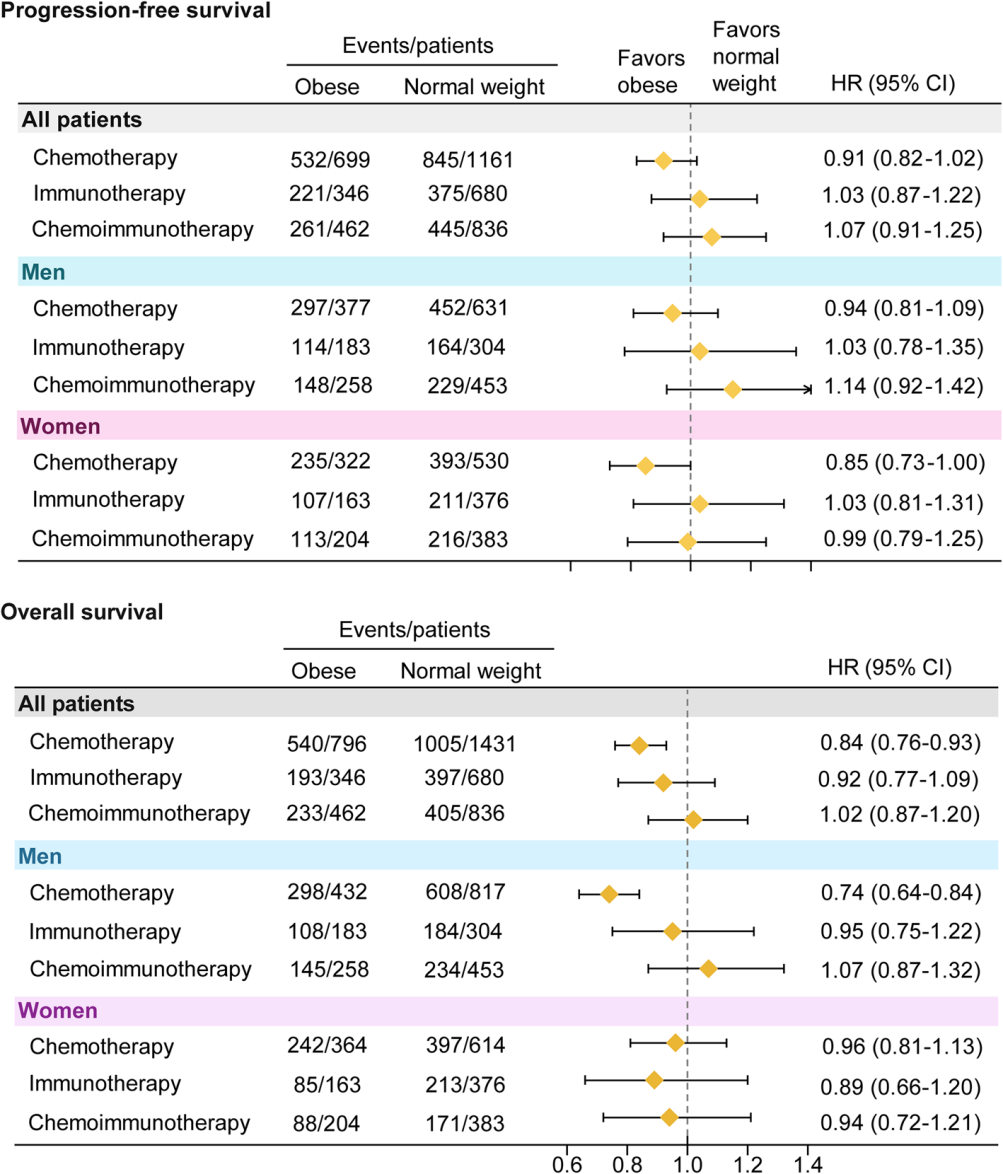
Pooled analysis. Forest plots of average adjusted hazard ratios (HRs) for patients with obese BMI compared with normal BMI by treatment type and sex for progression-free survival and overall survival

In the meta-analysis, obesity was not associated with improved PFS by treatment type in any patients or sex subgroups (Fig. 4). Although the association of obesity with OS for the combined immunotherapy and chemoimmunotherapy therapy cohorts was consistent with that for PFS, we found a significant association between obesity and OS benefit in chemotherapy patients (Fig. 4). The survival benefit difference was more pronounced for men (HR = 0.74; 95% CI 0.64–0.84) than women (HR = 0.96; 95% CI 0.81–1.13). More importantly, the difference in the association of BMI with OS by sex in the chemotherapy-treated cohort analysis reached statistical significance (P_interactio*n* =_ 0.018).

**Fig. 5:**
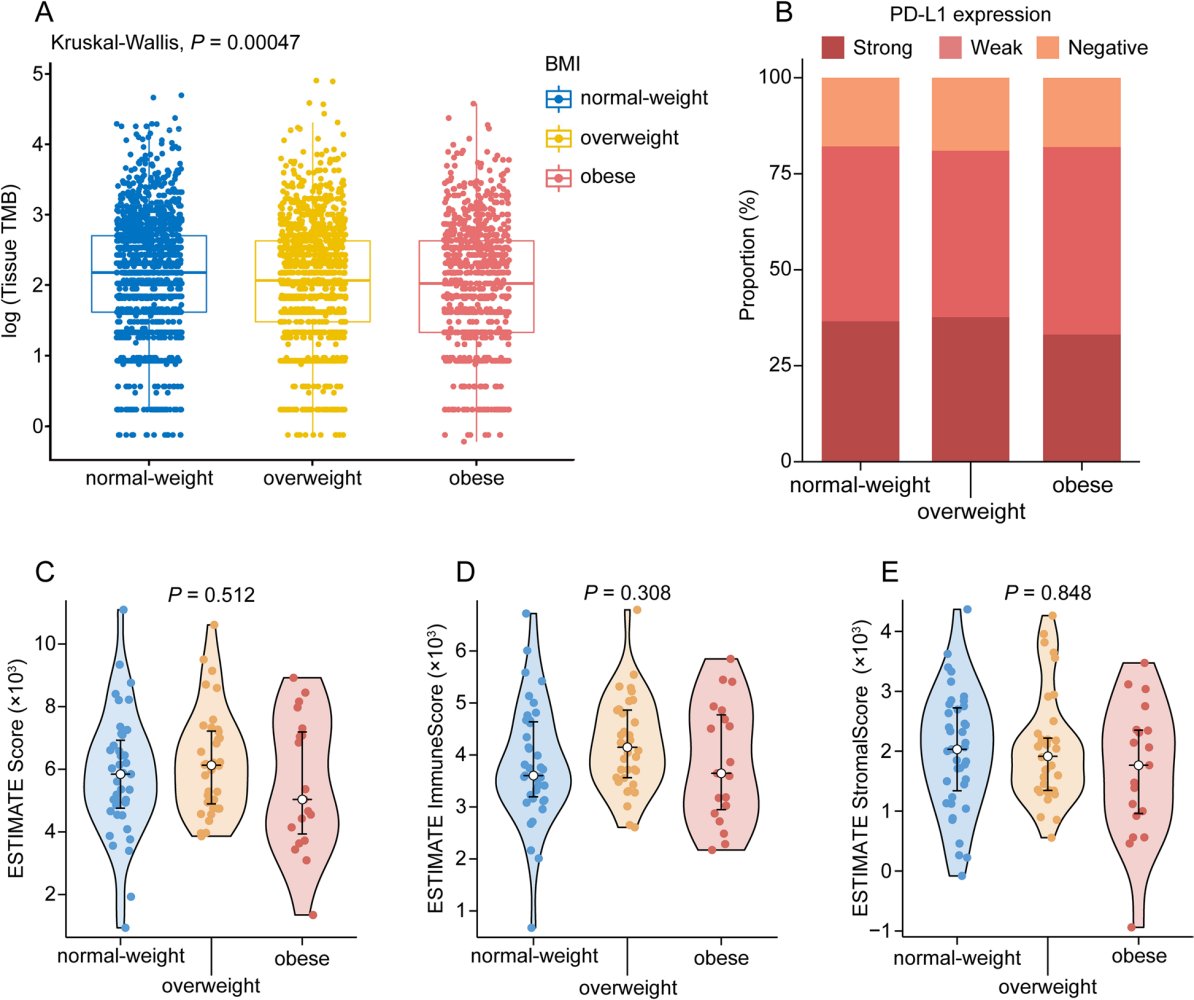
The level of biomarker by BMI category. Association between BMI and **A** tissue tumor mutational burden and **B** PD-L1 expression in CGDB cohorts. Association between BMI and **C** ESTIMATE score, **D** ESTIMATE Immune Score, and **E** ESTIMATE Stromal Score

We then explored the association between BMI and tumor mutation burden (TMB), programmed death-ligand 1 (PD-L1) expression, and ESTIMATE scores. Patients at normal weight had the highest level of TMB, while obese patients had the lowest level (Fig. 5A). No difference was observed between BMI and PD-L1 expression (Fig. 5B). Similarly, we found no significant association of BMI with ESTIMATE score, ESTIMATE Immune Score, or ESTIMATE Stromal Score (Figs. 5C-E).

## Discussion

In this retrospective study of 7021 patients with advanced NSCLC, we investigated the relationship between BMI and clinical outcomes. Our findings suggested that, compared with the patients at a normal weight, obese patients receiving chemotherapy have improved OS, but not PFS. This association varied by sex, with a survival advantage observed in men but not in women. In addition, no significant association between BMI and PFS or OS was found in patients treated with immunotherapy or chemoimmunotherapy.

Some large-scale studies have demonstrated an inverse association between BMI and risk of overall mortality for patients with NSCLC [2–4, 25], and most of the included patients received frontline chemotherapy. In addition, a pooled study (*n* = 2585) of ECOG5592, ECOG1594 and ECOG4599 found that obese patients had superior OS compared with normal/overweight patients (HR = 0.86; 95% CI 0.75–0.99) [7]. Our results were consistent with these studies (HR = 0.87; 95% CI 0.77–0.98). However, a recent study found no significant differences according to baseline BMI in the chemotherapy cohort in NSCLC patients with a PD-L1 expression ≥ 50% [5]. We also observed this result in our study with a limited sample size (*n* = 107, data not shown). Therefore, further studies are needed to determine the association between BMI and survival in patients with a high level of PD-L1 expression.

There are at least two explanations for the inverse relationship between obesity and the risk of death in patients treated with chemotherapy: First, obese patients may have increased rates of type 2 diabetes, hypertension, cardiovascular disease, and other co-morbid conditions [26], which may cause these patients to seek medical attention and receive optimal medical treatment, which would result in the reduced mortality risk. Second, high-grade toxicity from chemotherapy could lead to treatment-related weight loss, which has been demonstrated as an independent prognostic factor for survival of patients with NSCLC [27]. Several studies have suggested that obese patients may have greater physiologic reserve to counteract toxic effects from chemotherapy [28].

ASCO Guideline recommended that full, weight-based cytotoxic chemotherapy doses should be used to treat obese adults with cancer [29]. Therefore, obese patients may achieve better clinical outcomes. In this study, we noted that obese patients receiving chemotherapy had improved OS. However, there was only a marginal benefit in PFS (HR = 0.91; 95% CI 0.82–1.02). It was possible that the non-significance for this result was due to small sample size and lack of statistical power.

In the current study, we observed that the protective effect of obesity on OS was significantly greater in male patients than in female ones receiving chemotherapy. Similarly, in a pooled analysis of 20,937 NSCLC patients, Jiang et al. also found that male patients who were obese had relatively better OS (P_interactio*n* =_ 0.016) compared with obese female patients [25]. Endogenous estrogen may play a protective role against lung cancer death; in a large-scale prospective cohort study, the authors found that women who used oral contraceptives had a significantly higher risk of lung cancer death compared with never-users [30]. Among post-menopausal women, the risk increased by 2% with each year since menopause [30]. Obesity in men could lead to higher levels of circulating estradiol, because adipose tissue aromatase may convert androgens to estrogen compounds [31], which was confirmed in a recent study [32]. However, increased BMI was also associated with increased levels of estrogen in women [33]. Thus, the reasons for a potential protective effect of obesity in male patients remain unclear, and our findings must be cautiously interpreted. Future studies should further address whether and how obesity reduces death risk in men with NSCLC receiving chemotherapy.

Our study found no impact of obesity on outcomes in patients receiving immunotherapy or chemoimmunotherapy. The reasons for this association may be multifactorial. First, previous studies have reported that obesity was associated with the efficacy of ICIs in NSCLC [5, 34–36]. However, most of these studies included patients treated with second- or later-line PD-1/PD-L1 inhibitors. A recent study (*n* = 84) indicated no significant difference in the PFS (HR = 0.94; 95% CI 0.53–1.65) or OS (HR = 0.67; 95% CI 0.32–1.40) between patients with high and low BMI in first-line pembrolizumab therapy [6], which was consistent with our findings. Additionally, most of previous studies were retrospective, and the significant association might vanish after controlling for more confounders [37, 38]. Second, a recent study found a nonlinear association between BMI and OS following treatment with ICI in advanced NSCLC [39]. Risk of death increased at both extremes of BMI with a nadir at approximately 30 [39]. We also observed this nonlinear association in the current study (data not shown). Third, Ringel et al. demonstrated that obesity induced by a high-fat diet impaired CD8^+^T cell function in the murine tumor microenvironment [40]. Furthermore, in the current study obesity was not associated with higher levels of PD-L1 expression or ESTIMATE Immune Score, implying no association between obesity and a “hot” tumor microenvironment. Finally, we found that patients at normal weight had the highest level of TMB, suggesting that they might have higher levels of neoantigens. Chemotherapy can induce the release of neoantigens and promote the activity of cytotoxic T cells [41]. Therefore, chemoimmunotherapy might achieve synergistic effects in NSCLC patients with normal weight.

Oigometastatic disease (OMD) is an intermediary stage between localized disease and diffuse spread. A recent systematic review and meta-analysis found that PFS and OS improvements associated with the addition of local consolidative therapy (LCT) for metastatic NSCLC (largely for OMD) [42]. Therefore, it is interesting to assess the impact of BMI on LCT for metastatic NSCLC with OMD. This important clinical issue should be investigated in future studies.

This study has some limitations. First, most of the included cohorts were retrospective. Not all demographic and clinical variables could be captured. Thus, the possibility of residual confounding cannot be ruled out. Second, we should note the heterogeneity of the data came from seven cohorts. Clinical heterogeneity (such as variability in the participants) and methodological heterogeneity (such as variability in study design) existed in these seven cohorts. Therefore, the conclusions should be interpreted with caution. Third, we included only BMI value at baseline, and did not include dynamic changes in weight during treatment. Loss or gain in body weight was found to be significantly associated with survival in previous studies [3, 27, 43]. Fourth, because of dataset limitations, the data of ORR, toxicities and immune-related AE, which are critical for outcomes, were not included in all the cohorts. Finally, other anthropometric measures, such as waist circumference, waist-hip ratio, and visceral fat index, were not analyzed in this current study. Therefore, caution in interpretation of these results is necessary, and our results should be confirmed by future studies.

## Conclusions

In summary, obesity was associated with improved outcomes in male patients with NSCLC treated with chemotherapy, whereas obesity was not associated with outcomes in patients treated with first-line immunotherapy or chemoimmunotherapy. Future prospective studies with adjustment for more confounding factors are warranted before a conclusion can be drawn.

## Supplementary Information

Supplementary Material 1: Figure S1. Cubic spline graph of the HRand 95% CIfor the association between BMI and OS in NSCLC patients treated with chemotherapy in CGDB-Chemo cohort. Figure S2. Progression-free survival and overall survival by BMI category and sex in QL1101 cohort. Figure S3. Waterfall plot of the best response in NSCLC patients who received bevacizumab/QL1101 plus chemotherapy in normal weight, overweight, and obese population. Figure S4. Overall survival by BMI category and sex in SHChest-Chemo cohort. Figure S5. Progression-free survival and overall survival by BMI category and sex in Chowell-Immu cohort. Figure S6. Forest plots of PFS and OS for patients treated with penpulimab + chemotherapy compared with chemotherapy by BMI in AK105-302 cohort. Table S1. Baseline characteristics of the NSCLC patients receiving chemotherapy. Table S2. Baseline characteristics of the NSCLC patients receiving chemotherapy. Table S3. Response rates by BMI category and gender. Table S4. Baseline characteristics of the NSCLC patients receiving chemotherapy. Table S5. Baseline characteristics of the NSCLC patients receiving immunotherapy. Table S6. Baseline characteristics of the NSCLC patients. Table S7. Baseline characteristics of the NSCLC patients receiving chemoimmunotherapy. Table S8. Baseline characteristics of the NSCLC patients. Table S9. Adverse events by BMI.

## Data Availability

No datasets were generated or analysed during the current study.

## Abbreviations

ICI: Immune checkpoint inhibitor
BMI: Body mass index
PFS: Progression-free survival
OS: Overall survival
ICI: Immune checkpoint inhibitors
NSCLC: Non-small cell lung cancer
WHO: World Health Organization
RECIST: Response Evaluation Criteria in Solid Tumors
NGS: Next-generation sequencing
PD-L1: Programmed deathligand 1
ORR: Objective response rate
RCS: Restricted cubic spline
TMB: Tumor mutational burden
