# Supplementary material for: Obesity and survival in advanced non-small cell lung cancer patients treated with chemotherapy, immunotherapy, or chemoimmunotherapy: a multicenter cohort study
Source: BMC Med. 2024 Oct 14;22:463. doi: 10.1186/s12916-024-03688-2 (PMC11475647; doi:10.1186/s12916-024-03688-2)
Supplement: Supplementary file 1 — Supplementary Material 1: Figure S1. Cubic spline graph of the HRand 95% CIfor the association between BMI and OS in NSCLC patients treated with chemotherapy in CGDB-Chemo cohort. Figure S2. Progression-free survival and overall survival by BMI category and sex in QL1101 cohort. Figure S3. Waterfall plot of the best response in NSCLC patients who received bevacizumab/QL1101 plus chemotherapy in normal weight, overweight, and obese population. Figure S4. Overall survival by BMI category and sex in SHChest-Chemo cohort. Figure S5. Progression-free survival and overall survival by BMI category and sex in Chowell-Immu cohort. Figure S6. Forest plots of PFS and OS for patients treated with penpulimab + chemotherapy compared with chemotherapy by BMI in AK105-302 cohort. Table S1. Baseline characteristics of the NSCLC patients receiving chemotherapy. Table S2. Baseline characteristics of the NSCLC patients receiving chemotherapy. Table S3. Response rates by BMI category and gender. Table S4. Baseline characteristics of the NSCLC patients receiving chemotherapy. Table S5. Baseline characteristics of the NSCLC patients receiving immunotherapy. Table S6. Baseline characteristics of the NSCLC patients. Table S7. Baseline characteristics of the NSCLC patients receiving chemoimmunotherapy. Table S8. Baseline characteristics of the NSCLC patients. Table S9. Adverse events by BMI. [file 12916_2024_3688_MOESM1_ESM.docx]

**Eligibility criteria:**

**CGDB-Chemo, CGDB-Immu, and CGDB-Chemoimmu cohorts**

These three cohorts used deidentified clinical data from Flatiron Health-Foundation Medicine NSCLC clinico-genomic database (FH-FMI CGDB) and included advanced NSCLC patients treated with chemotherapy (combined with or without bevacizumab), immunotherapy, or chemoimmunotherapy as first-line treatment. Patients initiated therapy between 2003 and 2021.

**QL1101 cohort**

This cohort consisted of patients treated with chemotherapy plus bevacizumab/QL1101 on the randomized, double-blind clinical trial from November 1, 2016 and July 31, 2018. Patients from 54 centers in China were enrolled. Patients received chemotherapy plus bevacizumab/QL1101(15 mg/kg Q21d IV) until disease progression, death, unacceptable toxicity. The main inclusion criteria included: ages ≧ 18 years of age and < 75 years of age; pathologically-confirmed non-squamous stage IIIb or stage IV NSCLC; at least one measurable lesion; and an untreated or progressed disease and an Eastern Cooperative Oncology Group (ECOG) score of 0 or 1.

**SHChest-Chemo cohort**

This was a retrospective cohort of advanced NSCLC patients treated with chemotherapy (combined with or without bevacizumab) at Shanghai Chest Hospital. Patients initiated therapy between January 2012 and December 2015. Clinical characteristics were extracted including age, sex, stage, tumor type, smoking status, and *EGFR* mutation.

**Chowell-Immu cohort**

This cohort was derived from a published study (1,479 patients from 16 cancer types). We extracted 172 advanced NSCLC patients treated with frontline immunotherapy. Other clinical and genetic data, such as BMI, age, sex, loss of heterozygosity, neutrophil-to-lymphocyte ratio, hemoglobin, tumor mutational burden, fraction of copy number alteration, HLA-I evolutionary divergence, were provided by the study.

**AK105-302 cohort**

AK105-302 was a multicentre, randomised, double-blind, placebo-controlled phase 3 clinical trial. Patients on AK105-302 were randomized to penpulimab (anti-PD-1) + chemotherapy vs. placebo + chemotherapy at 74 centers in China between December 20, 2018 to October 10, 2020. The participants received penpulimab 200 mg or placebo plus paclitaxel 175 mg/m^2^ plus carboplatin (AUC 5) intravenously (IV) on d1 every 3 weeks (Q3W) for 4 cycles, followed by maintenance therapy with penpulimab 200 mg or placebo IV on d1 Q3W. Eligible patients were 18-75 years, histologically or cytologically confirmed locally advanced (stage IIIB/IIIC) or metastatic (stage IV) squamous NSCLC with measurable disease, ECOG PS of 0-1, not receive prior systemic therapy.


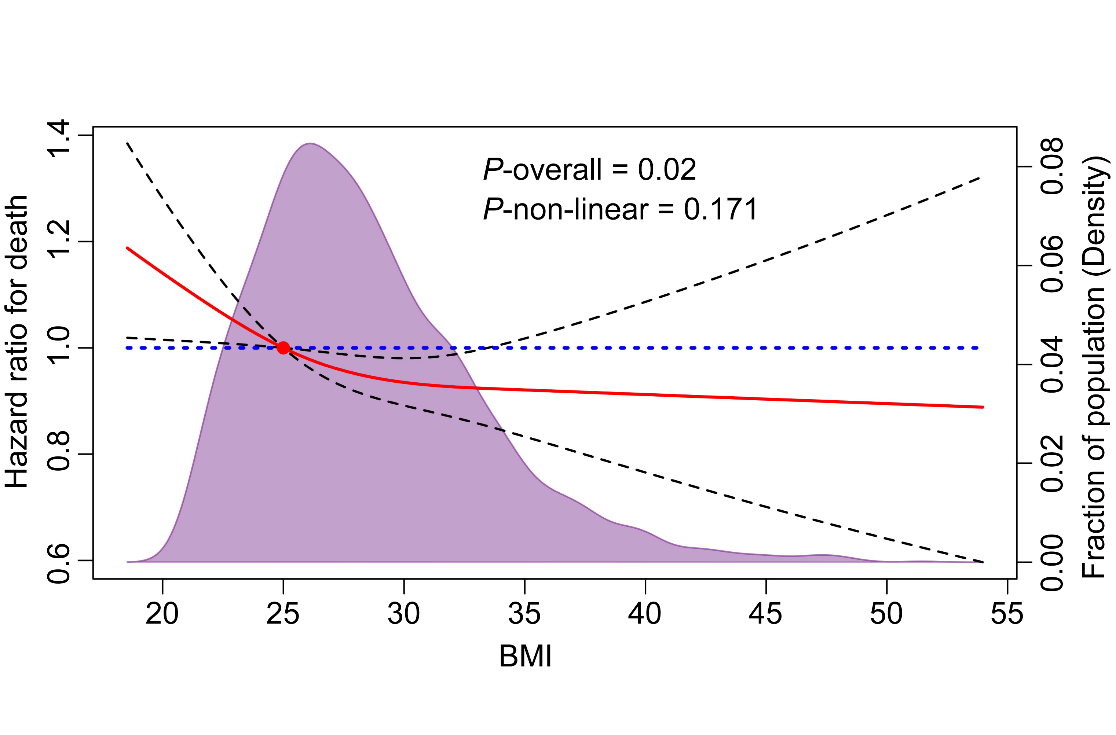


Figure S1. Cubic spline graph of the HR (represented by solid red line) and 95% CI (represented by the black dotted lines) for the association between BMI and OS in NSCLC patients treated with chemotherapy in CGDB-Chemo cohort. The purple area indicates the distribution of BMI. BMI=25 was chosen as reference.


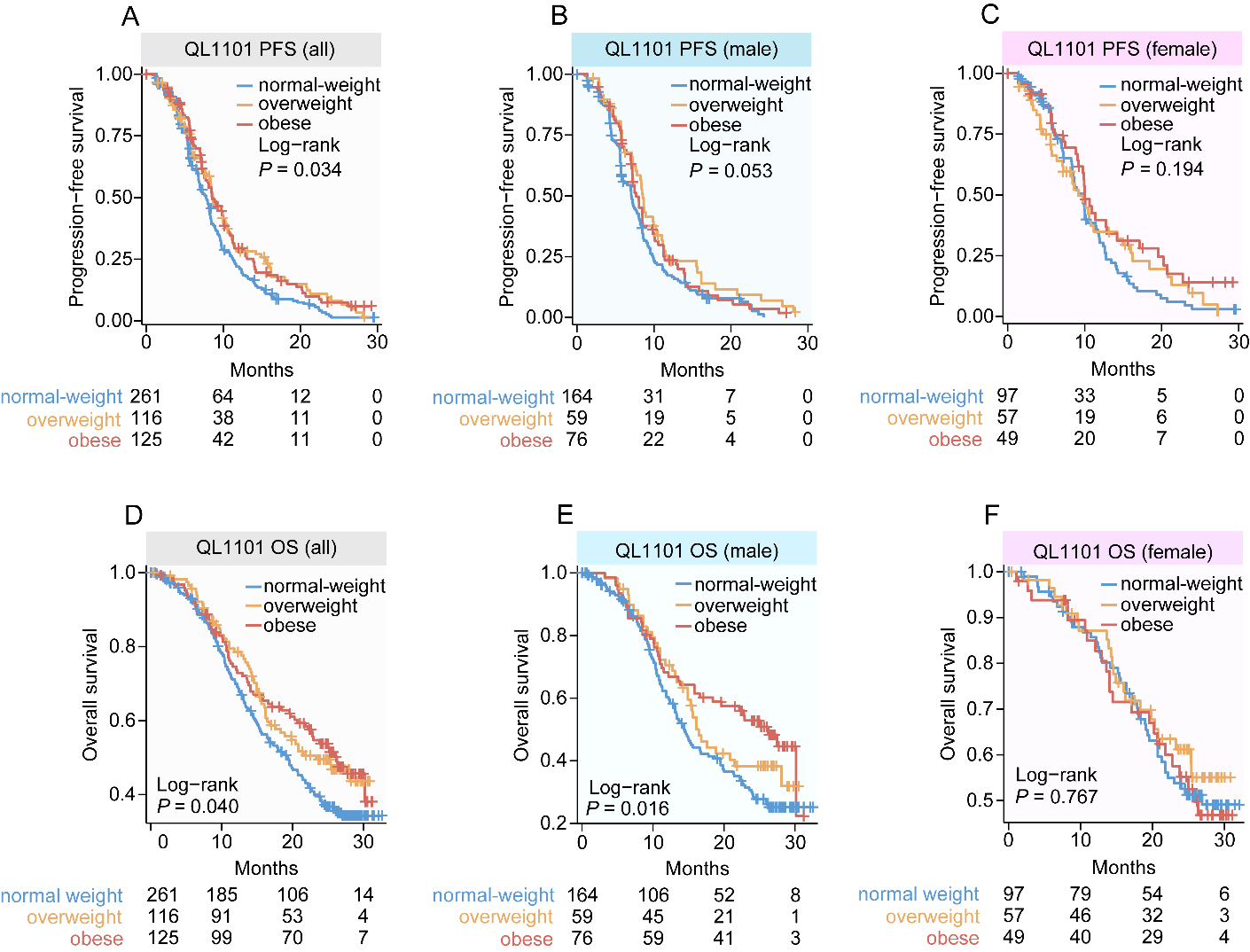


Figure S2. Progression-free survival and overall survival by BMI category and sex in QL1101 cohort.

Progression-free survival in the (A) overall patients, (B) male patients, (C) female patients. Overall survival in the (D) overall patients, (E) male patients, (F) female patients.


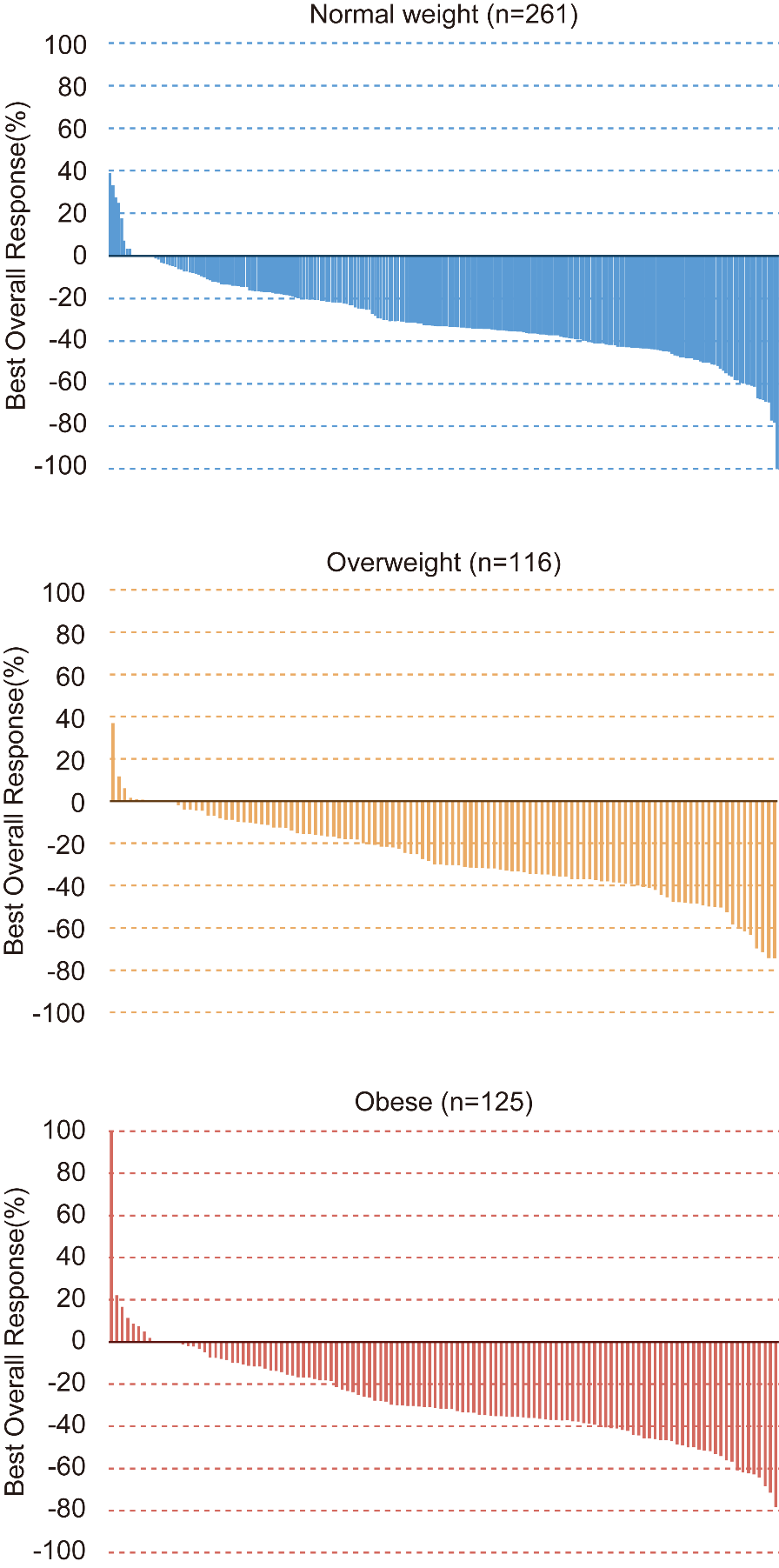


Figure S3. Waterfall plot of the best response in NSCLC patients who received bevacizumab/QL1101 plus chemotherapy in normal weight (up), overweight (middle), and obese population (down).


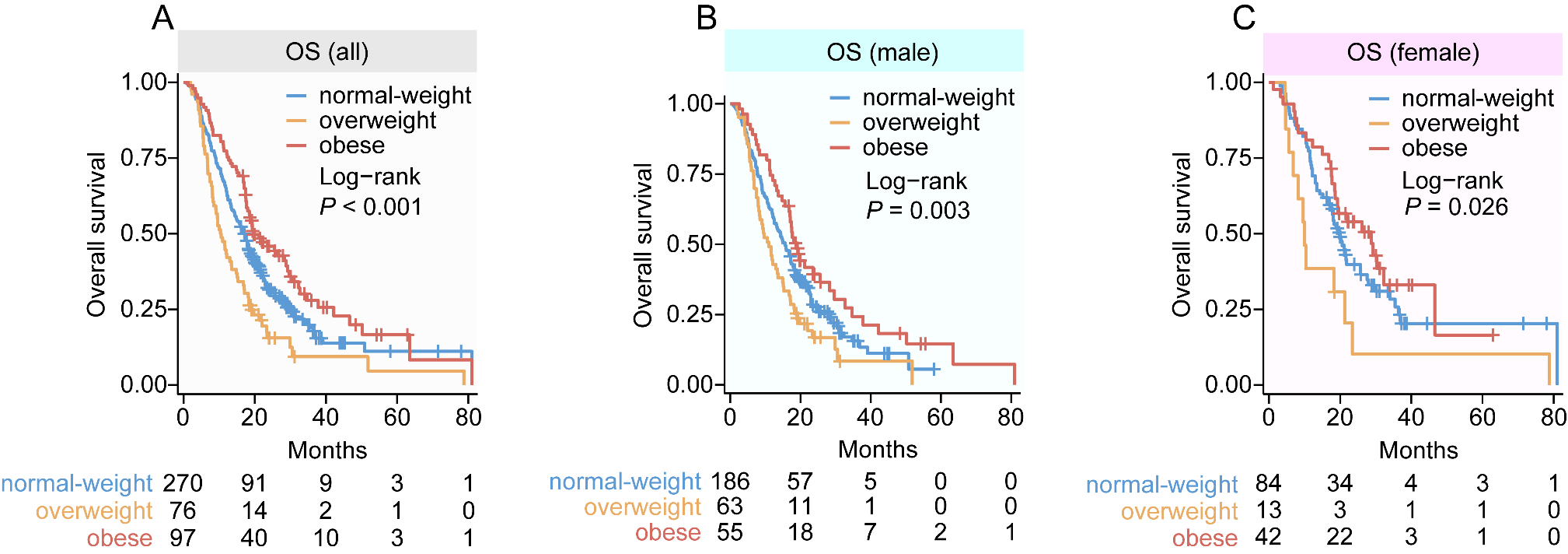


Figure S4. Overall survival by BMI category and sex in SHChest-Chemo cohort.

Overall survival in the (A) overall patients, (B) male patients, (C) female patients.


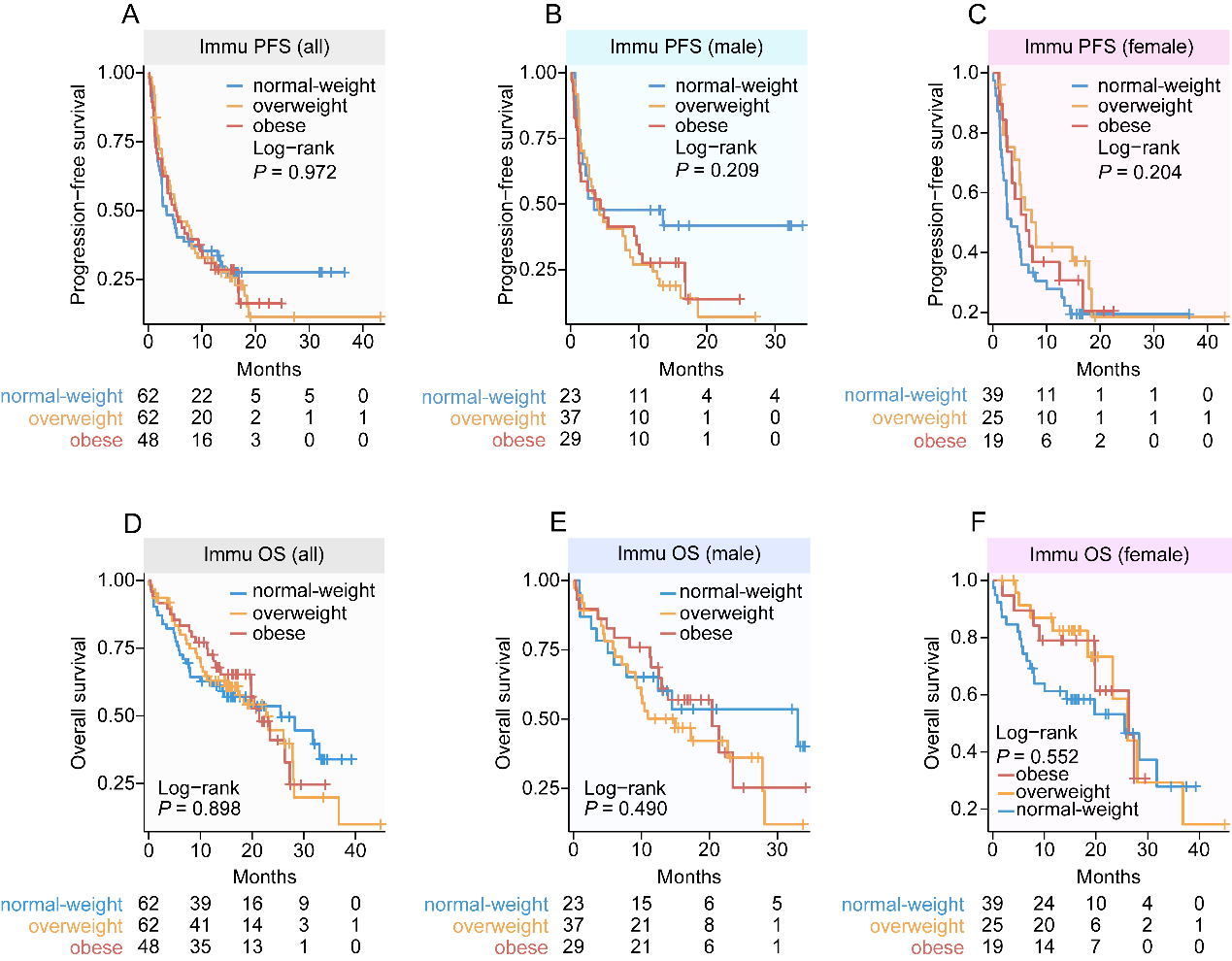


Figure S5. Progression-free survival and overall survival by BMI category and sex in Chowell-Immu cohort.

Progression-free survival in the (A) overall patients, (B) male patients, (C) female patients. Overall survival in the (D) overall patients, (E) male patients, (F) female patients.


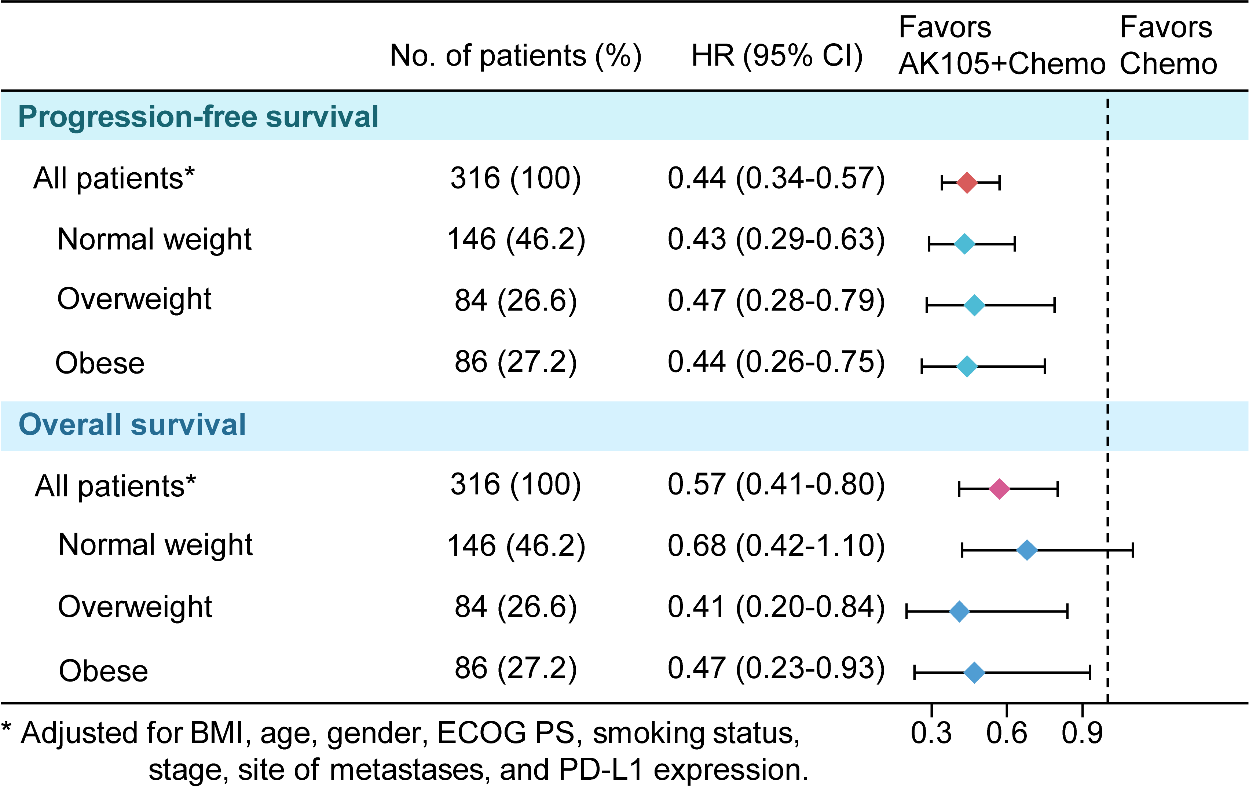


Figure S6. Forest plots of PFS and OS for patients treated with penpulimab + chemotherapy compared with chemotherapy by BMI in AK105-302 cohort.

Table S1. Baseline characteristics of the NSCLC patients receiving chemotherapy (CGDB-Chemo cohort)

|  | **Overall** | **Normal weight** | **Overweight** | **Obese** |
| --- | --- | --- | --- | --- |
|  | **(N=2246)** | **(N=902)** | **(N=770)** | **(N=574)** |
| **Median age (IQR)** | 74 (66-80) | 74 (66-80) | 75 (67-81) | 73 (66-79.8) |
| **Gender - no. (%)** |  |  |  |  |
| Female | 1015 (45.2) | 434 (48.1) | 308 (40.0) | 273 (47.6) |
| Male | 1231 (54.8) | 468 (51.9) | 462 (60.0) | 301 (52.4) |
| **Race - no. (%)** |  |  |  |  |
| White | 1685 (75.0) | 676 (74.9) | 575 (74.7) | 434 (75.6) |
| Black or African American | 177 (7.9) | 71 (7.9) | 52 (6.7) | 54 (9.4) |
| Other Race | 384 (17.1) | 155 (17.2) | 143 (18.6) | 86 (15) |
| **ECOG PS - no. (%)** |  |  |  |  |
| 0 | 751 (33.4) | 275 (30.5) | 287 (37.3) | 189 (32.9) |
| 1 | 1080 (48.1) | 453 (50.2) | 357 (46.4) | 270 (47.0) |
| >=2 | 415 (18.5) | 174 (19.3) | 126 (16.3) | 115 (20.1) |
| **Type of tumor - no. (%)** |  |  |  |  |
| Non-squamous | 1468 (65.4) | 584 (64.8) | 507 (65.8) | 377 (65.7) |
| Squamous | 686 (30.5) | 270 (29.9) | 235 (30.6) | 181 (31.5) |
| Other | 92 (4.1) | 48 (5.3) | 28 (3.6) | 16 (2.8) |
| **Smoking status - no. (%)** |  |  |  |  |
| History of smoking | 1987 (88.5) | 811 (89.9) | 671 (87.1) | 505 (88.0) |
| No history of smoking | 255 (11.4) | 90 (10.0) | 97 (12.6) | 68 (11.9) |
| Missing | 4 (0.1) | 1 (0.1) | 2 (0.3) | 1 (0.1) |
| **Stage - no. (%)** |  |  |  |  |
| IIIB/IIIC | 1015 (45.2) | 372 (41.2) | 361 (46.9) | 261 (45.4) |
| IV | 1231 (54.8) | 530 (58.8) | 409 (53.1) | 313 (54.5) |
| **Brain metastases - n (%)** |  |  |  |  |
| No | 1607 (71.5) | 627 (69.5) | 562 (73.0) | 418 (72.8) |
| Yes | 639 (28.5) | 275 (30.5) | 208 (27.0) | 156 (27.2) |
| **Bone metastases - n (%)** |  |  |  |  |
| No | 1364 (60.7) | 531 (58.9) | 464 (60.3) | 369 (64.3) |
| Yes | 882 (39.3) | 371 (41.1) | 306 (39.7) | 205 (35.7) |
| **Liver metastases - n (%)** |  |  |  |  |
| No | 1772 (78.9) | 697 (77.3) | 622 (80.8) | 453 (78.9) |
| Yes | 474 (21.1) | 205 (22.7) | 148 (19.2) | 121 (21.1) |
| ***EGFR* - no. (%)** |  |  |  |  |
| Negative | 858 (38.2) | 351 (38.9) | 308 (40.0) | 199 (34.7) |
| Positive | 53 (2.4) | 18 (2.0) | 21 (2.7) | 14 (2.4) |
| Missing | 1335 (59.4) | 533 (59.1) | 441 (57.3) | 361 (62.9) |
| ***ALK* - no. (%)** |  |  |  |  |
| Rearrangement | 9 (0.4) | 3 (0.3) | 4 (0.5) | 2 (0.4) |
| Negative | 819 (36.5) | 331 (36.7) | 293 (38.1) | 195 (34.0) |
| Missing | 1418 (63.1) | 568 (63.0) | 473 (61.4) | 377 (65.6) |
| ***ROS1* - no. (%)** |  |  |  |  |
| Rearrangement | 7 (0.3) | 4 (0.5) | 1 (0.1) | 2 (0.4) |
| Negative | 624 (27.8) | 243 (26.9) | 227 (29.5) | 154 (26.8) |
| Missing | 1615 (71.9) | 655 (72.6) | 542 (70.4) | 418 (72.8) |
| ***KRAS* - no. (%)** |  |  |  |  |
| Negative | 387 (17.2) | 161 (17.9) | 136 (17.7) | 90 (16.7) |
| Positive | 120 (5.3) | 47 (5.2) | 35 (4.5) | 38 (6.6) |
| Missing | 1739 (77.5) | 694 (76.9) | 599 (77.8) | 446 (77.7) |
| ***STK11* - no. (%)** |  |  |  |  |
| Negative | 4 (0.2) | 2 (0.2) | 1 (0.1) | 1 (0.2) |
| Positive | 307 (13.7) | 123 (13.6) | 102 (13.3) | 82 (14.3) |
| Missing | 1935 (86.1) | 777 (86.2) | 667 (86.6) | 491 (85.5) |
| ***KEAP1* - no. (%)** |  |  |  |  |
| Negative | 8 (0.4) | 3 (0.3) | 3 (0.4) | 2 (0.4) |
| Positive | 392 (17.5) | 152 (16.9) | 142 (18.4) | 98 (17.1) |
| Missing | 1846 (82.1) | 747 (82.8) | 625 (81.2) | 474 (82.5) |
| **Median tTMB (mut/megabase)** |  |  |  |  |
| Median (range) | 8.8 (4.4-14.0) | 8.8 (4.8-14.9) | 8.8 (4.4-13.2) | 8.8 (3.8-14.9) |
| <10 | 722 (32.2) | 269 (29.9) | 269 (34.9) | 184 (32.1) |
| >=10 | 561 (25.0) | 236 (26.1) | 188 (24.4) | 137 (23.9) |
| Missing | 963 (42.8) | 397 (44.0) | 313 (40.7) | 253 (44.0) |
| **Median bTMB (mut/megabase)** |  |  |  |  |
| Median (range) | 3.79 (1.26-7.59) | 5.06 (1.26-8.85) | 3.79 (2.85-7.59) | 2.53 (1.26-3.16) |
| <10 | 72 (3.2) | 37 (4.1) | 18 (2.3) | 17 (3.0) |
| >=10 | 18 (0.8) | 12 (1.3) | 4 (0.5) | 2 (0.3) |
| Missing | 2156 (96.0) | 853 (94.6) | 748 (97.1) | 555 (96.7) |
| **PD-L1 expression - no. (%)** |  |  |  |  |
| <1% | 81 (3.6) | 42 (4.7) | 17 (2.1) | 22 (3.8) |
| 1%<=PDL1<50% | 237 (10.6) | 102 (11.3) | 76 (9.9) | 59 (10.3) |
| >=50% | 107 (4.8) | 43 (4.8) | 39 (5.1) | 25 (4.4) |
| Missing | 1821 (81.0) | 715 (79.2) | 638 (82.9) | 468 (81.5) |

IQR, interquartile range; ECOG PS, Eastern Cooperative Oncology Group Performance Status; TMB, tumor mutational burden; PD-L1, programmed death-ligand 1

Table S2. Baseline characteristics of the NSCLC patients receiving chemotherapy (QL1101 cohort)

|  | **Overall** | **Normal weight** | **Overweight** | **Obese** |
| --- | --- | --- | --- | --- |
|  | **(N=502)** | **(N=261)** | **(N=116)** | **(N=125)** |
| **Median age (IQR)** | 58 (51-64) | 59 (52-64) | 57.5 (49-65) | 58 (50-64) |
| **Gender - no. (%)** |  |  |  |  |
| Female | 203 (40.4) | 97 (37.2) | 57 (49.1) | 49 (39.2) |
| Male | 299 (59.6) | 164 (62.8) | 59 (50.9) | 76 (60.8) |
| **ECOG PS - no. (%)** |  |  |  |  |
| 0 | 121 (24.1) | 44 (16.9) | 33 (28.4) | 44 (35.2) |
| 1 | 381 (75.9) | 217 (83.1) | 83 (71.6) | 81 (64.8) |
| **Smoking status - no. (%)** |  |  |  |  |
| History of smoking | 240 (47.8) | 139 (53.3) | 48 (41.4) | 53 (42.4) |
| No history of smoking | 262 (52.2) | 122 (46.7) | 68 (58.6) | 72 (57.6) |
| **Stage - no. (%)** |  |  |  |  |
| IIIB | 20 (4.0) | 8 (3.1) | 4 (3.4) | 8 (6.4) |
| IV | 482 (96.0) | 253 (96.9) | 112 (96.6) | 117 (93.6) |
| **Brain metastases - n (%)** |  |  |  |  |
| No | 472 (94.0) | 240 (92.0) | 114 (98.3) | 118 (94.4) |
| Yes | 30 (6.0) | 21 (8.0) | 2 (1.7) | 7 (5.6) |
| **Bone metastases - n (%)** |  |  |  |  |
| No | 498 (99.2) | 258 (98.9) | 116 (100.0) | 124 (100.0) |
| Yes | 4 (0.8) | 3 (1.1) | 0 (0) | 0 (0) |
| **Liver metastases - n (%)** |  |  |  |  |
| No | 462 (92.0) | 232 (88.9) | 110 (94.8) | 120 (96.0) |
| Yes | 40 (8.0) | 29 (11.1) | 6 (5.2) | 5 (4.0) |
| ***EGFR* - no. (%)** |  |  |  |  |
| Negative | 305 (60.8) | 157 (60.2) | 64 (55.2) | 84 (67.2) |
| Positive | 197 (39.2) | 104 (39.8) | 52 (44.8) | 41 (32.8) |

IQR, interquartile range; ECOG PS, Eastern Cooperative Oncology Group Performance Status

Table S3. Response rates by BMI category and gender

| Cohort | BMI | ORR | OR (95% CI) | P value |
| --- | --- | --- | --- | --- |
| QL1101 | | | | |
| All patients | Normal weight | 62.3% | 1 (ref) |  |
|  | Overweight | 53.6% | 0.70 (0.44-1.01) | 0.127 |
|  | Obese | 55.4% | 0.75 (0.48-1.17) | 0.206 |
| Male | Normal weight | 62.9% | 1 (ref) |  |
|  | Overweight | 50.9% | 0.61 (0.33-1.13) | 0.118 |
|  | Obese | 58.7% | 0.84 (0.47-1.48) | 0.538 |
| Female | Normal weight | 61.4% | 1 (ref) |  |
|  | Overweight | 56.6% | 0.82 (0.41-1.64) | 0.577 |
|  | Obese | 50.0% | 0.63 (0.31-1.29) | 0.208 |
| Chowell-Immu | | | | |
| All patients | Normal weight | 33.9% | 1 (ref) |  |
|  | Overweight | 40.3% | 1.32 (0.64-2.74) | 0.458 |
|  | Obese | 43.8% | 1.52 (0.70-3.30) | 0.291 |
| Male | Normal weight | 34.8% | 1 (ref) |  |
|  | Overweight | 32.4% | 0.90 (0.30-2.70) | 0.851 |
|  | Obese | 41.4% | 1.32 (0.43-4.11) | 0.628 |
| Female | Normal weight | 33.3% | 1 (ref) |  |
|  | Overweight | 52.0% | 2.17 (0.77-6.06) | 0.141 |
|  | Obese | 47.4% | 1.80 (0.59-5.52) | 0.304 |
| AK105-302 (Chemoimmu arm) | | | | |
| All patients | Normal weight | 66.7% | 1 (ref) |  |
|  | Overweight | 71.4% | 1.25 (0.56-2.79) | 0.586 |
|  | Obese | 77.1% | 1.68 (0.72-3.92) | 0.228 |
| Male | Normal weight | 66.7% | 1 (ref) |  |
|  | Overweight | 72.1% | 1.29 (0.55-3.02) | 0.554 |
|  | Obese | 75.0% | 1.50 (0.64-3.55) | 0.356 |
| AK105-302 (Chemo arm) | | | | |
| All patients | Normal weight | 45.0% | 1 (ref) |  |
|  | Overweight | 37.1% | 0.72 (0.32-1.63) | 0.434 |
|  | Obese | 39.5% | 0.80 (0.36-1.75) | 0.572 |
| Male | Normal weight | 44.7% | 1 (ref) |  |
|  | Overweight | 30.0% | 0.53 (0.22-1.31) | 0.529 |
|  | Obese | 40.0% | 0.82 (0.37-1.86) | 0.640 |

BMI, body mass index; ORR, objective response rate; OR, odds ratio.

Table S4. Baseline characteristics of the NSCLC patients receiving chemotherapy (SHChest-Chemo cohort)

|  | **Overall** | **Normal weight** | **Overweight** | **Obese** |  |
| --- | --- | --- | --- | --- | --- |
|  | **(N=443)** | **(N=270)** | **(N=76)** | **(N=97)** |  |
| **Median age (IQR)** | 59 (53-65) | 59 (53-65) | 62 (54.5-66) | 55 (50-62) |  |
| **Gender - no. (%)** |  |  |  |  |  |
| Female | 139 (31.4) | 84 (31.1) | 13 (17.1) | 42 (56.7) |  |
| Male | 304 (68.6) | 186 (68.9) | 63 (82.9) | 55 (56.7) |  |
| **Type of tumor - no. (%)** |  |  |  |  |  |
| Non-squamous | 313 (70.7) | 186 (68.9) | 50 (65.8) | 77 (79.4) |  |
| Squamous | 130 (29.3) | 84 (31.1) | 26 (34.2) | 20 (20.6) |  |
| **Smoking status - no. (%)** |  |  |  |  |  |
| History of smoking | 221 (49.9) | 140 (51.9) | 49 (64.5) | 32 (33.0) |  |
| No history of smoking | 222 (50.1) | 130 (48.1) | 27 (35.5) | 65 (67.0) |  |
| **Stage - no. (%)** |  |  |  |  |  |
| IIIB/IIIC | 153 (34.5) | 97 (35.9) | 23 (30.3) | 33 (34.0) |  |
| IV | 290 (65.5) | 173 (64.1) | 53 (69.7) | 64 (66.0) |  |
| **ECOG PS - no. (%)** |  |  |  |  |  |
| 0 | 168 (37.9) | 104 (38.6) | 32 (42.1) | 32 (33.0) |  |
| 1 | 186 (42.0) | 110 (40.7) | 27 (35.5) | 49 (50.5) |  |
| >=2 | 89 (20.1) | 56 (20.7) | 17 (22.4) | 16 (16.5) |  |
| ***EGFR* - no. (%)** |  |  |  |  |  |
| Negative | 85 (19.2) | 44 (16.3) | 11 (14.5) | 30 (30.9) |  |
| Positive | 358 (80.8) | 226 (83.7) | 65 (85.5) | 67 (69.1) |  |

IQR, interquartile range; ECOG PS, Eastern Cooperative Oncology Group Performance Status

Table S5. Baseline characteristics of the NSCLC patients receiving immunotherapy (CGDB-Immu cohort)

|  | **Overall** | **Normal weight** | **Overweight** | **Obese** |  |
| --- | --- | --- | --- | --- | --- |
|  | **(N=1386)** | **(N=618)** | **(N=470)** | **(N=298)** |  |
| **Median age (IQR)** | 75 (68-81) | 76 (68-81) | 76.5 (68-83) | 73 (66-79) |  |
| **Gender - no. (%)** |  |  |  |  |  |
| Female | 695 (50.1) | 337 (54.5) | 214 (45.5) | 144 (48.3) |  |
| Male | 691 (49.9) | 281 (45.5) | 256 (54.5) | 154 (51.7) |  |
| **Race - no. (%)** |  |  |  |  |  |
| White | 1053 (76.0) | 476 (77.0) | 356 (75.7) | 221 (74.2) |  |
| Black or African American | 92 (6.6) | 34 (5.5) | 35 (7.5) | 23 (7.7) |  |
| Other Race | 241 (17.4) | 108 (17.5) | 79 (16.8) | 54 (18.1) |  |
| **ECOG PS - no. (%)** |  |  |  |  |  |
| 0 | 374 (27.0) | 162 (26.2) | 136 (28.9) | 76 (25.5) |  |
| 1 | 685 (49.4) | 299 (48.4) | 229 (48.7) | 157 (52.7) |  |
| >=2 | 327 (23.6) | 157 (25.4) | 105 (22.4) | 65 (21.8) |  |
| **Type of tumor - no. (%)** |  |  |  |  |  |
| Non-squamous | 943 (68.0) | 413 (66.8) | 328 (69.8) | 202 (67.8) |  |
| Squamous | 386 (27.9) | 175 (28.3) | 126 (26.8) | 85 (28.5) |  |
| Other | 57 (4.1) | 30 (4.9) | 16 (3.4) | 11 (3.7) |  |
| **Smoking status - no. (%)** |  |  |  |  |  |
| History of smoking | 1286 (92.8) | 577 (93.4) | 433 (92.1) | 276 (92.6) |  |
| No history of smoking | 99 (7.1) | 41 (6.6) | 36 (7.7) | 22 (7.4) |  |
| Missing | 1 (0.1) | 0 (0) | 1 (0.2) | 0 (0) |  |
| **Stage - no. (%)** |  |  |  |  |  |
| IIIB/IIIC | 478 (34.5) | 198 (32.0) | 162 (34.5) | 118 (39.6) |  |
| IV | 908 (65.5) | 420 (68.0) | 308 (65.5) | 180 (60.4) |  |
| **Brain metastases - n (%)** |  |  |  |  |  |
| No | 1033 (74.5) | 461 (74.6) | 345 (73.4) | 227 (76.2) |  |
| Yes | 353 (25.5) | 157 (25.4) | 125 (26.6) | 71 (23.8) |  |
| **Bone metastases - n (%)** |  |  |  |  |  |
| No | 842 (60.8) | 363 (58.7) | 291 (61.9) | 188 (63.1) |  |
| Yes | 544 (39.2) | 255 (41.3) | 179 (38.1) | 110 (36.9) |  |
| **Liver metastases - n (%)** |  |  |  |  |  |
| No | 1127 (81.3) | 508 (82.2) | 384 (81.7) | 235 (78.9) |  |
| Yes | 259 (18.7) | 110 (17.8) | 86 (18.3) | 63 (21.1) |  |
| ***EGFR* - no. (%)** |  |  |  |  |  |
| Negative | 543 (39.2) | 249 (40.3) | 168 (35.7) | 126 (42.3) |  |
| Positive | 16 (1.2) | 5 (0.8) | 5 (1.1) | 6 (2.0) |  |
| Missing | 827 (59.6) | 364 (58.9) | 297 (63.2) | 166 (55.7) |  |
| ***ALK* - no. (%)** |  |  |  |  |  |
| Rearrangement | 2 (0.1) | 0 (0) | 1 (0.2) | 1 (0.3) |  |
| Negative | 514 (37.1) | 233 (37.7) | 166 (35.3) | 115 (38.6) |  |
| Missing | 870 (62.8) | 385 (62.3) | 303 (64.5) | 182 (61.1) |  |
| ***ROS1* - no. (%)** |  |  |  |  |  |
| Rearrangement | 2 (0.1) | 1 (0.2) | 0 (0) | 1 (0.3) |  |
| Negative | 470 (33.9) | 217 (35.1) | 146 (31.1) | 107 (35.9) |  |
| Missing | 914 (66.0) | 400 (64.7) | 324 (69.0) | 190 (63.8) |  |
| ***KRAS* - no. (%)** |  |  |  |  |  |
| Negative | 228 (16.5) | 103 (16.7) | 70 (14.9) | 55 (18.5) |  |
| Positive | 115 (8.3) | 51 (8.3) | 33 (7.0) | 31 (10.4) |  |
| Missing | 1043 (75.2) | 464 (75.0) | 367 (78.1) | 212 (71.1) |  |
| ***STK11* - no. (%)** |  |  |  |  |  |
| Negative | 4 (0.3) | 3 (0.5) | 0 (0) | 1 (0.3) |  |
| Positive | 166 (12.0) | 76 (12.3) | 50 (10.6) | 40 (13.4) |  |
| Missing | 1216 (87.7) | 539 (87.2) | 420 (89.4) | 257 (86.3) |  |
| ***KEAP1* - no. (%)** |  |  |  |  |  |
| Negative | 9 (0.7) | 5 (0.8) | 1 (0.2) | 3 (1.0) |  |
| Positive | 221 (16.0) | 102 (16.5) | 81 (17.2) | 38 (12.8) |  |
| Missing | 1156 (83.3) | 511 (82.7) | 388 (82.6) | 257 (86.2) |  |
| **Median tTMB (mut/megabase)** |  |  |  |  |  |
| Median (range) | 8.8 (5.0-14.0) | 8.8 (5.0-13.9) | 8.8 (5.0-15.1) | 8.8 (5.0-13.9) |  |
| <10 | 563 (40.6) | 257 (41.6) | 180 (38.3) | 126 (42.3) |  |
| >=10 | 476 (34.3) | 220 (35.6) | 162 (34.5) | 94 (31.5) |  |
| Missing | 347 (25.1) | 141 (22.8) | 128 (27.2) | 78 (26.2) |  |
| **Median bTMB (mut/megabase)** |  |  |  |  |  |
| Median (range) | 5.1 (2.5-10.1) | 5.7 (2.5-12.3) | 5.1 (2.5-8.9) | 2.5 (1.3-7.6) |  |
| <10 | 56 (4.1) | 24 (3.9) | 19 (4.0) | 13 (4.4) |  |
| >=10 | 20 (1.4) | 10 (1.6) | 6 (1.3) | 4 (1.3) |  |
| Missing | 1310 (94.5) | 584 (94.5) | 445 (94.7) | 281 (94.3) |  |
| **PD-L1 - no. (%)** |  |  |  |  |  |
| <1% | 41 (3.0) | 15 (2.4) | 17 (3.6) | 9 (3.1) |  |
| 1%<=PDL1<50% | 156 (11.2) | 64 (10.4) | 49 (10.4) | 43 (14.4) |  |
| >=50% | 320 (23.1) | 155 (25.1) | 100 (21.3) | 65 (21.8) |  |
| Missing | 869 (62.7) | 384 (62.1) | 304 (64.7) | 181 (60.7) |  |

IQR, interquartile range; ECOG PS, Eastern Cooperative Oncology Group Performance Status; TMB, tumor mutational burden; PD-L1, programmed death-ligand 1

Table S6. Baseline characteristics of the NSCLC patients (Chowell-Immu cohort)

|  | **Overall** | **Normal weight** | **Overweight** | **Obese** |
| --- | --- | --- | --- | --- |
|  | **(N=172)** | **(N=62)** | **(N=62)** | **(N=48)** |
| **Median age (IQR)** | 65.4 (59.8-72.8) | 65.4 (59.5-74.4) | 64.3 (58.3-72.3) | 67.6 (60.8-72.0) |
| **Gender - no. (%)** |  |  |  |  |
| Female | 83 (48.3) | 39 (62.9) | 25 (40.3) | 19 (39.6) |
| Male | 89 (51.7) | 23 (37.1) | 37 (59.7) | 29 (60.4) |
| **ECOG PS - no. (%)** |  |  |  |  |
| 0 | 65 (36.8) | 18 (29.0) | 25 (40.3) | 22 (45.8) |
| 1 | 99 (57.6) | 41 (66.1) | 34 (54.8) | 24 (50.0) |
| >=2 | 8 (4.7) | 3 (4.8) | 3 (4.8) | 2 (4.2) |
| **LOH - no. (%)** |  |  |  |  |
| Yes | 41 (23.8) | 14 (22.6) | 15 (24.2) | 12 (25.0) |
| No | 131 (76.2) | 48 (77.4) | 47 (75.8) | 36 (75.0) |
| **Median NLR (IQR)** | 4.6 (2.9-6.1) | 4.9 (3.1-8.1) | 4.4 (2.8-5.5) | 4.4 (2.9-5.7) |
| **Median platelets (IQR)** | 299 (239.5-375.8) | 329.0 (242.8-446.3) | 300.0 (248.8-376.0) | 262.0 (202.5-32.5) |
| **Median HGB (IQR)** | 12.5 (11.4-13.6) | 11.9 (11.0-12.9) | 12.4 (11.6-13.4) | 13.3 (12.4-14.1) |
| **Median albumin (IQR)** | 3.8 (3.5-4.1) | 3.8 (3.5-4.0) | 3.9 (3.7-4.2) | 3.9 (3.5-4.2) |
| **Median TMB (IQR)** | 7.0 (3.5-11.4) | 8.8 (4.3-12.9) | 7.0 (3.5-11.0) | 6.0 (3.5-11.4) |
| **Median FCNA (IQR)** | 0.1 (0.02-0.3) | 0.1 (0.02-0.3) | 0.1 (0.01-0.3) | 0.1 (0.03-0.3) |
| **Median HED (IQR)** | 6.6 (5.5-7.9) | 6.6 (5.6-7.8) | 6.6 (5.4-7.8) | 6.5 (5.7-8.1) |
| **Treatment- no. (%)** |  |  |  |  |
| Combo | 33 (19.2) | 11 (17.7) | 9 (14.5) | 13 (27.1) |
| Mono | 139 (80.8) | 51 (82.3) | 53 (85.5) | 35 (72.9) |

LOH, loss of heterozygosity; NLR, neutrophil-to-lymphocyte ratio; HGB, hemoglobin; TMB, tumor mutational burden; FCNA, fraction of copy number alteration; HED, HLA-I evolutionary divergence; Combo, anti-PD-1/PD-L1 plus anti-CTLA-4; Mono, anti-PD-1, abti-PD-L1, or anti-CTLA-4.

Table S7. Baseline characteristics of the NSCLC patients receiving chemoimmunotherapy (CGDB-Chemoimmu cohort)

|  | **Overall** | **Normal weight** | **Overweight** | **Obese** |  |
| --- | --- | --- | --- | --- | --- |
|  | **(N=1956)** | **(N=836)** | **(N=658)** | **(N=462)** |  |
| **Median age (IQR)** | 72 (65-78) | 72 (64-78) | 72 (65-78) | 71.5 (65-76) |  |
| **Gender - no. (%)** |  |  |  |  |  |
| Female | 852 (43.6) | 383 (45.8) | 265 (40.3) | 204 (44.2) |  |
| Male | 1104 (56.4) | 453 (51.2) | 393 (59.7) | 258 (55.8) |  |
| **Race - no. (%)** |  |  |  |  |  |
| White | 1444 (73.8) | 602 (72.0) | 487 (74.0) | 355 (76.8) |  |
| Black or African American | 136 (7.0) | 57 (6.8) | 43 (6.5) | 36 (7.8) |  |
| Other Race | 376 (19.2) | 177 (21.2) | 128 (19.5) | 71 (15.4) |  |
| **ECOG PS - no. (%)** |  |  |  |  |  |
| 0 | 723 (37.0) | 306 (36.6) | 249 (37.8) | 168 (36.4) |  |
| 1 | 892 (45.6) | 384 (46.0) | 298 (45.3) | 210 (45.4) |  |
| >=2 | 341 (17.4) | 146 (17.4) | 111 (16.9) | 84 (18.2) |  |
| **Type of tumor - no. (%)** |  |  |  |  |  |
| Non-squamous | 1422 (72.7) | 610 (73.0) | 486 (73.9) | 326 (70.6) |  |
| Squamous | 451 (23.1) | 186 (22.3) | 148 (22.4) | 117 (25.3) |  |
| Other | 83 (4.2) | 40 (4.7) | 24 (3.7) | 19 (4.1) |  |
| **Smoking status - no. (%)** |  |  |  |  |  |
| History of smoking | 1774 (90.7) | 766 (91.6) | 597 (90.7) | 411 (89.0) |  |
| No history of smoking | 182 (9.3) | 70 (8.4) | 61 (9.3) | 51 (11.0) |  |
| Missing | 0 (0) | 0 (0) | 0 (0) | 0 (0) |  |
| **Stage - no. (%)** |  |  |  |  |  |
| IIIB/IIIC | 651 (33.3) | 258 (30.9) | 219 (33.3) | 174 (37.7) |  |
| IV | 1305 (66.7) | 578 (69.1) | 439 (66.7) | 288 (62.3) |  |
| **Brain metastases - n (%)** |  |  |  |  |  |
| No | 1450 (74.1) | 600 (71.8) | 494 (75.1) | 356 (77.1) |  |
| Yes | 506 (25.9) | 236 (28.2) | 164 (24.9) | 106 (22.9) |  |
| **Bone metastases - n (%)** |  |  |  |  |  |
| No | 1120 (57.3) | 459 (54.9) | 384 (58.4) | 277 (60.0) |  |
| Yes | 836 (42.7) | 377 (45.1) | 274 (41.6) | 185 (40.0) |  |
| **Liver metastases - n (%)** |  |  |  |  |  |
| No | 1605 (82.1) | 694 (83.0) | 532 (80.9) | 379 (82.0) |  |
| Yes | 351 (17.9) | 142 (17.0) | 126 (19.1) | 83 (18.0) |  |
| ***EGFR* - no. (%)** |  |  |  |  |  |
| Negative | 682 (34.9) | 282 (33.7) | 234 (35.6) | 166 (35.9) |  |
| Positive | 23 (1.1) | 7 (0.8) | 12 (1.8) | 4 (0.9) |  |
| Missing | 1251 (64.0) | 547 (65.5) | 412 (62.6) | 292 (63.2) |  |
| ***ALK* - no. (%)** |  |  |  |  |  |
| Rearrangement | 3 (0.2) | 1 (0.1) | 0 (0) | 2 (0.4) |  |
| Negative | 667 (34.1) | 275 (32.9) | 236 (35.9) | 156 (33.8) |  |
| Missing | 1286 (65.7) | 560 (67.0) | 422 (64.1) | 304 (65.8) |  |
| ***ROS1* - no. (%)** |  |  |  |  |  |
| Rearrangement | 3 (0.1) | 2 (0.2) | 1 (0.2) | 0 (0) |  |
| Negative | 663 (33.9) | 263 (31.5) | 241 (36.6) | 159 (34.4) |  |
| Missing | 1290 (66.0) | 571 (68.3) | 416 (63.2) | 303 (65.6) |  |
| ***KRAS* - no. (%)** |  |  |  |  |  |
| Negative | 370 (19.0) | 143 (17.1) | 140 (21.3) | 87 (18.8) |  |
| Positive | 146 (7.4) | 57 (6.8) | 45 (6.8) | 44 (9.5) |  |
| Missing | 1440 (73.6) | 636 (76.1) | 473 (71.9) | 331 (71.7) |  |
| ***STK11* - no. (%)** |  |  |  |  |  |
| Negative | 6 (0.3) | 4 (0.5) | 1 (0.2) | 1 (0.2) |  |
| Positive | 347 (17.7) | 151 (18.1) | 118 (17.9) | 78 (16.9) |  |
| Missing | 1603 (82.0) | 681 (81.4) | 539 (81.9) | 383 (82.9) |  |
| ***KEAP1* - no. (%)** |  |  |  |  |  |
| Negative | 5 (0.2) | 2 (0.2) | 3 (0.5) | 0 (0) |  |
| Positive | 357 (18.3) | 154 (18.4) | 116 (17.6) | 87 (18.8) |  |
| Missing | 1594 (81.5) | 680 (81.4) | 539 (81.9) | 375 (81.2) |  |
| **Median tTMB (mut/megabase)** |  |  |  |  |  |
| Median (range) | 7.6 (3.8-12.6) | 7.9 (3.8-13.9) | 7.6 (3.8-12.6) | 6.3 (3.8-11.4) |  |
| <10 | 954 (48.8) | 386 (46.2) | 334 (50.8) | 234 (50.7) |  |
| >=10 | 604 (30.9) | 292 (34.9) | 185 (28.1) | 127 (27.5) |  |
| Missing | 398 (20.3) | 158 (18.9) | 139 (21.1) | 101 (21.8) |  |
| **Median bTMB (mut/megabase)** |  |  |  |  |  |
| Median (range) | 3.8 (1.3-10.1) | 6.3 (1.6-12.6) | 3.8 (1.3-5.7) | 3.2 (1.3-8.5) |  |
| <10 | 117 (6.0) | 44 (5.3) | 46 (7.0) | 27 (5.8) |  |
| >=10 | 42 (2.2) | 26 (3.1) | 9 (1.4) | 7 (1.5) |  |
| Missing | 1797 (91.8) | 766 (91.6) | 603 (91.6) | 428 (92.7) |  |
| **PD-L1 expression - no. (%)** |  |  |  |  |  |
| <1% | 99 (5.1) | 36 (4.3) | 43 (6.5) | 20 (4.3) |  |
| 1%<=PDL1<50% | 275 (14.1) | 126 (15.1) | 76 (11.5) | 73 (15.8) |  |
| >=50% | 159 (8.1) | 70 (8.4) | 53 (8.1) | 36 (7.8) |  |
| Missing | 1423 (72.7) | 604 (72.2) | 486 (73.9) | 333 (72.1) |  |

IQR, interquartile range; ECOG PS, Eastern Cooperative Oncology Group Performance Status; TMB, tumor mutational burden; PD-L1, programmed death-ligand 1

Table S8. Baseline characteristics of the NSCLC patients (AK105-302 cohort)

|  | **Overall** | **Normal weight** | **Overweight** | **Obese** |
| --- | --- | --- | --- | --- |
|  | **(N=316)** | **(N=146)** | **(N=84)** | **(N=86)** |
| **Median age (IQR)** | 62 (55-66) | 62 (57-66) | 60 (55-65) | 61 (55-66) |
| **Gender - no. (%)** |  |  |  |  |
| Female | 25 (7.9) | 7 (4.8) | 11 (13.1) | 7 (8.1) |
| Male | 291 (92.1) | 139 (95.2) | 73 (86.9) | 79 (91.9) |
| **ECOG PS - no. (%)** |  |  |  |  |
| 0 | 74 (23.4) | 33 (22.6) | 21 (25.0) | 20 (23.3) |
| 1 | 242 (76.6) | 113 (77.4) | 63 (75.0) | 66 (76.7) |
| **Smoking status - no. (%)** |  |  |  |  |
| History of smoking | 277 (87.7) | 131 (89.7) | 71 (84.5) | 75 (87.2) |
| No history of smoking | 39 (12.3) | 15 (10.3) | 13 (15.5) | 11 (12.8) |
| **Stage - no. (%)** |  |  |  |  |
| IIIB/IIIC | 43 (13.6) | 18 (12.3) | 13 (15.5) | 12 (14.0) |
| IV | 273 (86.4) | 128 (87.7) | 71 (84.5) | 74 (86.0) |
| **Brain metastases - n (%)** |  |  |  |  |
| No | 307 (97.2) | 140 (95.9) | 83 (98.8) | 84 (97.7) |
| Yes | 9 (2.8) | 6 (4.1) | 1 (1.2) | 2 (2.3) |
| **Bone metastases - n (%)** |  |  |  |  |
| No | 244 (77.2) | 114 (78.1) | 65 (77.4) | 65 (75.6) |
| Yes | 72 (22.8) | 32 (21.9) | 19 (22.6) | 21 (24.4) |
| **Liver metastases - n (%)** |  |  |  |  |
| No | 277 (87.7) | 125 (85.6) | 73 (86.9) | 79 (91.9) |
| Yes | 39 (12.3) | 21 (14.4) | 11 (13.1) | 7 (8.1) |
| **PD-L1 expression - no. (%)** |  |  |  |  |
| <1% | 106 (33.7) | 50 (34.2) | 28 (33.7) | 28 (32.6) |
| 1%<=PDL1<50% | 150 (47.6) | 72 (49.3) | 39 (47.0) | 39 (45.3) |
| >=50% | 59 (18.7) | 24 (16.4) | 16 (19.3) | 19 (22.1) |
| **Treatment- no. (%)** |  |  |  |  |
| Penpulimab + Chemotherapy | 163 (51.6) | 66 (45.2) | 49 (58.3) | 48 (55.8) |
| Chemotherapy | 153 (48.4) | 80 (54.8) | 35 (41.7) | 38 (44.2) |

IQR, interquartile range; ECOG PS, Eastern Cooperative Oncology Group Performance Status; PD-L1, programmed death-ligand 1

Table S9. Adverse events by BMI

| Population | BMI | Patient  No. (%) | Adverse events | | | Immune-related adverse events | | |
| --- | --- | --- | --- | --- | --- | --- | --- | --- |
|  |  |  | Grade I-II  No. (%) | Grade III  No. (%) | Grade IV-V  No. (%) | Grade I-II  No. (%) | Grade III  No. (%) | Grade IV-V  No. (%) |
| QL1101 | Normal weight | 261 (52.0) | 154 (59.0) | 21 (8.0) | 1 (0.4) | -- | -- | -- |
|  | Overweight | 116 (23.1) | 63 (54.3) | 9 (7.8) | 0 (0) | -- | -- | -- |
|  | Obese | 125 (24.9) | 78 (62.4) | 15 (12.0) | 0 (0) | -- | -- | -- |
| AK105-302 (Chemoimmu arm) | Normal weight | 66 (40.7) | 64 (97.0) | 40 (60.6) | 27 (40.9) | 17 (25.8) | 4 (6.1) | 2 (3.0) |
|  | Overweight | 50 (30.9) | 50 (100.0) | 36 (72.0) | 21 (42.0) | 15 (30.0) | 3 (6.0) | 0 (0.0) |
|  | Obese | 46 (28.4) | 46 (100.0) | 26 (56.5) | 15 (32.6) | 9 (19.6) | 1 (2.2) | 0 (0.0) |
| AK105-302 (Chemo arm) | Normal weight | 80 (52.3) | 80 (100.0) | 56 (70.0) | 33 (41.3) | -- | -- | -- |
|  | Overweight | 36 (23.5) | 36 (100.0) | 23 (63.9) | 12 (33.3) | -- | -- | -- |
|  | Obese | 37 (24.2) | 36 (97.3) | 22 (59.5) | 16 (43.2) | -- | -- | -- |

BMI, body mass index
